# Combining Transcriptome and Whole Genome Re-Sequencing to Screen Disease Resistance Genes for Wheat Dwarf Bunt

**DOI:** 10.3390/ijms242417356

**Published:** 2023-12-11

**Authors:** Yufeng Jia, Tong Shen, Zhiwei Wen, Jing Chen, Qi Liu

**Affiliations:** 1 Key Laboratory of Prevention and Control of Invasive Alien Species in Agriculture & Forestry of the North-Western Desert Oasis, Ministry of Agriculture and Rural Affairs, Urumqi 830052, China; 13899651356@163.com (Y.J.); 18331519385@163.com (T.S.); wen19945842141@163.com (Z.W.); chenj@xjau.edu.cn (J.C.); 2 Key Laboratory of the Pest Monitoring and Safety Control of Crops and Forests of the Xinjiang Uygur, Autonomous Region, College of Agronomy, Xinjiang Agricultural University, Urumqi 830052, China; 3 College of Plant Protection, Nanjing Agricultural University, Nanjing 210095, China

**Keywords:** Wheat dwarf bunt, *Tilletia controversa* Kühn, transcriptome, whole genome re-sequencing, disease resistance gene

## Abstract

Wheat dwarf bunt is a damaging disease caused by *Tilletia controversa* Kühn (TCK). Once the disease infects wheat, it is difficult to control and will significantly reduce wheat output and quality. RNA sequencing and whole genome re-sequencing were used to search for potential TCK resistance genes in Yili 053 (sensitive variety) and Zhongmai 175 (moderately resistant variety) in the mid-filling, late-filling, and maturity stages. The transcriptomic analysis revealed 11 potential disease resistance genes. An association analysis of the findings from re-sequencing found nine genes with single nucleotide polymorphism mutations. The Kyoto Encyclopedia of Genes and Genomes enrichment analysis showed that three up-regulated genes were involved in the synthesis of benzoxazinone and tryptophan metabolism. Additionally, quantitative real-time polymerase chain reaction confirmed the RNA sequencing results. The results revealed novel TCK resistance genes and provide a theoretical basis for researching the function of resistance genes and molecular breeding.

## 1. Introduction

Wheat (*Triticum*) is an important grain crop in the world [1]. Wheat dwarf bunt (WDB) caused by *Tilletia controversa* Kühn (TCK) is one of the major diseases of wheat. WDB significantly reduces wheat production and quality, which may decline by 20% to 80% [2,3]. TCK is a serious international disease that is now under quarantine [4]. Trade between China and other countries is expanding, and the TCK introduction risk is rising along with it, which potentially poses a threat to Chinese wheat production [5]. Winter wheat is the primary host of TCK, which may infect more than 70 species of plants in 18 genera of the Gramineae family [6]. Wheat generates a large number of tillers after infecting TCK, and its height is only 1/3 to 2/3 of healthy plants [7]. The infected wheat grains are replaced by black teliospores, which are called “bunt balls”, creating a distinct rotten fish odor due to trimethylamine [8]. Bunt balls are a serious primary infection source because they may survive in the soil for a long time [9]. WDB can spread through soil and seeds, and prevention and control efforts are still challenging. At present, the most economical, effective, and efficient technique for preventing WDB is to select varieties with significant resistance genes. Research on screening disease-resistant germplasm resources is underway, and there are few reports about WDB resistance materials in China; therefore, it is very important to accelerate the discovery of resistance genes tightly linked with TCK and take precautions to prevent the introduction of TCK from spreading.

There is still a serious lack of knowledge about the genetic basis of WDB resistance. There are currently 16 race-specific bunt resistance genes, including Bt1 to Bt15 and BtP, of which only two Bt genes (Bt9 and Bt10) have been mapped to specific chromosomal regions [10], and 24 quantitative trait loci (QTL) for bunt resistance have been identified by QTL mapping in wheat [11]. Conventional breeding was used to identify target genes associated with the phenotype of disease resistance, but it had the drawbacks of being time-consuming, labor-intensive, expensive, and ineffective. Therefore, WDB resistance breeding in wheat has been hampered by the excavation of functional genes related to the resistance of WDB.

With the development of high-throughput sequencing technology, whole genome re-sequencing (WGRS) and RNA sequencing (RNA-Seq) have been successfully used in plants, such as rice [12,13], chickpea [14], soybean [15], and wheat [16,17], and represent powerful tools for obtaining candidate genes associated with disease-resistant traits and genome characterization during the interaction of pathogens and plants, making breeding high throughput, inexpensive and more efficient. The whole genome level differences between individuals or groups can be examined using the WGRS, which involves the genomes of species with known genome sequences. WGRS is commonly used to identify genetic polymorphisms because it makes it simple to identify a large number of altered genes, such as single nucleotide polymorphisms (SNPs) and small insertions and deletions (InDels) [18]. The discovery of functional SNPs or InDels will greatly accelerate the process and efficiency of molecular marker-assisted breeding in plants. RNA-Seq is also called transcriptome sequencing; this technology can efficiently describe the dynamics and quantity of gene expression in plants under a specific situation [19]. The gene expression profile of plant interactions with pathogens may be thoroughly examined using RNA-Seq, such as mining resistance genes relevant to plant diseases and analyzing the corresponding resistance mechanisms.

In the current study, we evaluated the responses of two wheat varieties at three periods following TCK infection in order to learn more about genome variation at the molecular level in different resistant materials. Therefore, changes in the genome and gene expression in wheat varieties impacted by pathogens may be completely explored in depth based on these huge amounts of sequencing data.

The objective of this study was to identify candidate WDB-resistant genes in two varieties of wheat, Yili 053 (sensitive variety) and Zhongmai 175 (moderately resistant variety), respectively. RNA-Seq and WGRS were performed to analyze the changes in gene expression and the genome in response to TCK infection at the mid-filling stage, late-filling stage, and maturity stage, which helped us to explored candidate WDB resistance genes in the two wheat varieties. Meanwhile, this study will provide an important reference to excavate potential molecular markers and molecular design breeding related to WDB disease resistance in wheat.

## 2. Results

### 2.1. Whole Genome Re-Sequencing Analysis

The two varieties generated 632.80 Gbp of clean data after filtering. The clean data from Zhongmai 175 were 1,013,423,192 with a 16× average depth, and the Q30 ratio was 93.61. The clean data from Yili 053 were 1,098,471,243 with a 17× average depth, and the Q30 ratio was 93.72 (Table S1), which indicated that the sequencing data were high quality. There were 99.52% sequences on average that were aligned to the reference genome (Table S2), suggesting that our re-sequenced genomes are closely related to the reference genome.

**Figure 1:**
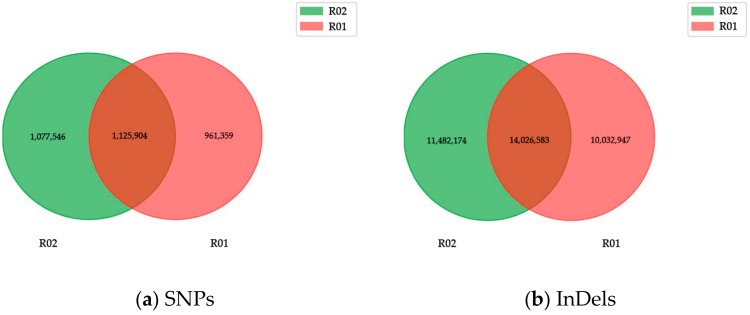
Venn diagram of genetic variation among samples. Note: R01 represents Zhongmai175 and R02 represents Yili053.

Zhongmai175 included 24,085,517 SNPs in total, of which 71.01% were transitions and 28.99% were transversions, and 83.7% were homozygous and 16.3% were heterozygous. Additionally, Yili053 had 25,534,555 SNPs, of which 71.03% were transitions and 28.97% were transversions, and 84.17% were homozygous and 15.8% were heterozygous. The transition/transversion ratio was detected, with 2.45 for Yili053 and 2.44 for Zhongmai175. The heterozygosity of SNPs in Zhongmai175 was higher than in Yili053, and the distribution of the SNPs is shown in (Table S3, Figure S1). Zhongmai175 had 15,079 InDels in the CDS region (47.44% insertions, 52.56% deletions), and 2,203,974 in the genome (48.08% insertions, 51.92% deletions). A total of 14,692 InDels were found in the Yili053 CDS region (47.73% insertions, 52.27% deletions), while 2,318,474 were found in the genome (48.27% insertions, 51.73% deletions). (Table S4). Most of the InDels (insertions and deletions) were found to be short InDels (1–3 bp) and longer than 10 bp in the CDS region, while those 1–2 bp in length were detected in the genome (Figure S2). Short InDels may have deleterious effects on gene functionality during expression or transcriptional processes [20]. In general, Yili053 had a higher number of detected SNPs and InDels than Zhongmai175 (Table 1), which were visualized in the wheat chromosomes using the Circos Visualization tool (Figure S3). Meanwhile, we analyzed the results to find the common and distinct genes of SNP and InDel variations between the two varieties (Figure 1). The results indicated that Zhongmai175 and Yili053 included 961,359 and 1,077,546 distinct SNPs. Additionally, there were 10,032,947 distinct InDels in Zhongmai175 and 11,482,174 in Yili053, respectively.

The detected SNPs and InDels were annotated using SnpEff software (v4.1) to evaluate the distribution and possible effects on the two varieties. In Zhongmai175 and Yili053, the ratio of SNPs located in the intergenic region was the highest (90.74%, 90.97%), followed by upstream region (3.79%, 3.68%), downstream region (3.26%, 3.19%), introns (1.12%, 1.11%), and CDS region (0.87%, 0.83%), as well as in the CDS region with non-synonymous coding (49.19%, 48.96%) and synonymous coding (49.88%, 50.12%) variants, respectively. Likewise, there were 81.16% and 81.53% InDels in intergenic regions, followed by the upstream region (7.62%, 7.46%), downstream region (6.55%, 6.44%), introns (3.04%, 3.02%), CDS region (0.71%, 0.66%). Moreover, in the CDS region, there were frame shifts (56.31%, 55.53%), codon insertions (14.89%, 15.39%), and codon deletions (12.89%, 12.73) (Figure S4). Frameshift mutations in InDel mutations may result in changes in gene function [21]. The majority of genomic variations were found in non-coding regions, and fewer were found in coding regions.

### 2.2. RNA Sequencing Analysis

RNA-Seq analysis was performed as an additional method to investigate changes in gene expression and find the candidate genes between diseased and healthy wheat grains from the two varieties in different stages after TCK inoculation. A total of 424.18 GB of clean data were acquired after filtering raw reads and deleting low-quality reads. Each sample’s clean data totaled 9.76 GB, the Q30 percentage was from 90.77% to 95.46%, and the GC contents of all the identified bases ranged between 51.8% and 58.83% (Table S5).

**Figure 2:**
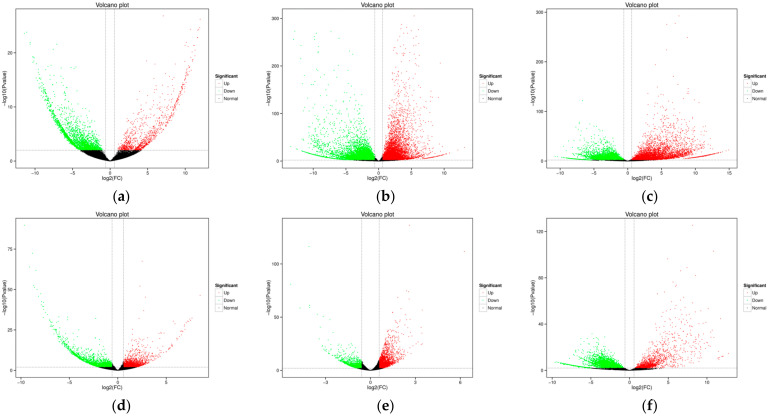
The overall distribution of DEGs in the two varieties at three growth periods. (**a**) Comparison groups: YLC and YLI in the mid-filling stage; (**b**) YLC and YLI in the late-filling stage; (**c**) YLC and YLI in the maturity stage; (**d**) ZMC and ZMI in the mid-filling stage; (**e**) ZMC and ZMI in the late-filling stage; (**f**) ZMC and ZMI in the maturity stage. Group nomenclatures are shown in materials and methods. The horizontal axis shows the change in the expression levels of DEGs in different samples; the vertical axis shows the statistical significance of the change inexpression levels. Red dots represent genes that are up-regulated and green dots mean genes that are down-regulated.

Differentially expressed genes (DEGs) in the two varieties at three growth periods were identified according to the FPKM value of every gene. As a result, 5413 (2710 up-regulated genes, 2703 down-regulated genes), 4914 (2987 up-regulated genes, 1930 down-regulated genes) and 13,417 (1752 up-regulated genes, 11,665 down-regulated genes) DEGs were identified at mid-filling stage, late-filling stage and maturity stage in Zhongmai175. Meanwhile, 3930 (1148 up-regulated genes, 2782 down-regulated genes), 28,422 (11405 up-regulated genes, 17,017 down-regulated genes) and 20,874 (7374 up-regulated genes, 13,500 down-regulated genes) DEGs were identified at the mid-filling stage, late-filling stage and maturity stage in Yili35 (Table S6). The overall distributions of DEGs with different expression levels in the two varieties were shown in (Figure 2). After TCK infection in Zhongmai175, the quantity of DEGs gradually decreased before abruptly increasing. The up-regulated genes increased during the final stages of filling, while declining during the maturation stage. Additionally, at the maturation phase, the down-regulated genes increased more than in the late filling stages. In contrast to resistant materials, the amount of DEGs in Yili35 increased initially and later declined after TCK infection. During the late-filling phase, the up-regulated and down-regulated genes both gradually increased, and declined during the maturation phase. Overall, there were 2.24 times more DEGs in Yili053 than in Zhongmai175. In other words, compared to resistant material, susceptible material required higher differential gene expression in response to TCK infection.

### 2.3. Functional Annotation of DEGs

In order to screen the resistance-related genes in response to TCK, sequence alignments were performed to annotate the predicted functions of these DEGs with four protein databases in six different DEG sets (group 1: I-YLC vs. I-YLI; group 2: I-ZMC vs. I-ZMI; group 3: II-YLC vs. II-YLI; group 4: II-ZMC vs. II-ZMI; group 5: III-YLC vs. III-YLI; group 6: III-ZMC vs. III-ZMI). The statistical DEG functional annotation classification is shown in Table 2. According to the GO enrichment analysis, all DEGs in the six groups were categorized into three major functional groups: biological processes, cellular components and molecular functions. The most representative terms in biological processes were metabolic processes, cellular processes, and single-organism processes; those in cellular components were membrane, membrane part, cell, cell part, and organelle; and those in molecular functions were binding and catalytic activity in the six groups (Figure S5).

Clusters of Orthologous Groups (COG) could analyze the evolution of different expressed genes and predict the functions of proteins in the genome (Figure S6). In Yili053, the gene functions were involved in carbohydrate transport and metabolism, post-translational modification, protein turnover, and chaperones during the mid-filling stage, while during the late-filling stage and maturity stage, they were carbohydrate transport and metabolism, translation, ribosomal structure, biogenesis, post-translational modification, protein turnover, chaperones, and signal transduction mechanisms. In Zhongmai175, the DEG functions were mainly divided into carbohydrate transport and metabolism, and signal transduction mechanisms during the mid-filling stages. The late-filling stage involved carbohydrate transport and metabolism, secondary metabolite biosynthesis, transport, and catabolism. Translation, ribosomal structure, biogenesis, post-translational modification, protein turnover, and chaperones were involved during the maturity stage.

**Figure 3:**
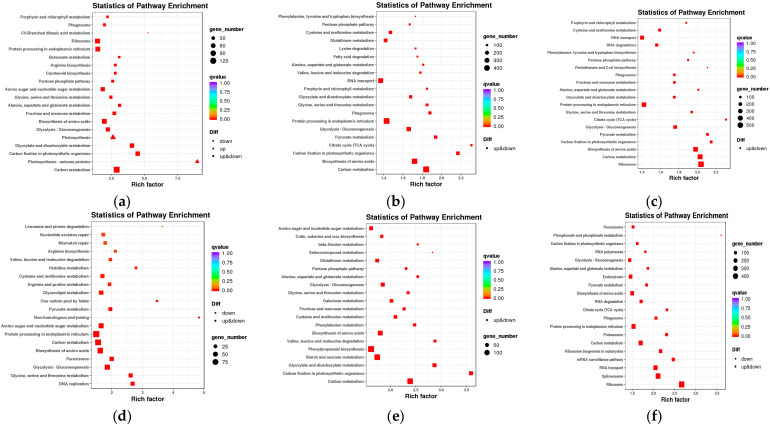
KEGG enrichment analysis of DE genes in the two varieties at three growth periods. (**a**) YLC and YLI in the mid-filling stage; (**b**) YLC and YLI in the late-filling stage; (**c**) YLC and YLI in the maturity stage; (**d**) ZMC and ZMI in the mid-filling stage; (**e**) ZMC and ZMI in the late-filling stage; (**f**) ZMC and ZMI in the maturity stage. Group nomenclatures are shown in in materials and methods. The horizontal axis represents the gene ratio; the vertical axis represents the KEGG pathways enriched for DEGs.

A Kyoto Encyclopedia of Genes and Genomes (KEGG) analysis was used to understand the biological functions of genes and determine the participation of DEGs in important signal transduction and metabolic pathways. The pathways with the highest number of DEGs in the top 20 are shown in Figure 3.

The metabolic pathways in Yili053 that had the greatest number of enhanced genes—121 DEGs—were those related to carbon metabolism during the mid-filing stage. The photosynthetic antenna protein pathway had the highest enrichment factor (8.98), suggesting that DEGs were the most enriched in this metabolic pathway. Carbon metabolism had the largest gene enrichment (480 DEGs) during the late-filing stage, while the citrate cycle (TCA cycle) had the highest enrichment factor value (2.31). The mature stage contained ribosomes with 525 DEGs, with the TCA cycle being the most highly enriched factor (2.31).

Protein processing in the endoplasmic reticulum in the mid-filing stage was found to be significantly enriched in Zhongmai175 (93 DEGs), while non-homologous end-joining had the highest value at 4.84. Phenylpropanoid biosynthesis contained the most genes (140 DEGs) during the late-filing stage, while carbon fixation in photosynthetic organisms had the highest enrichment factor value (3.57). Ribosomes had the most enhanced genes (489 DEGs) during the mature stage, phosphonate metabolism had the highest enrichment factor of 3.6. The annotation of DEGs’ projected functions gave more knowledge that was needed to continue this research.

### 2.4. Transcription Factor Prediction

**Figure 4:**
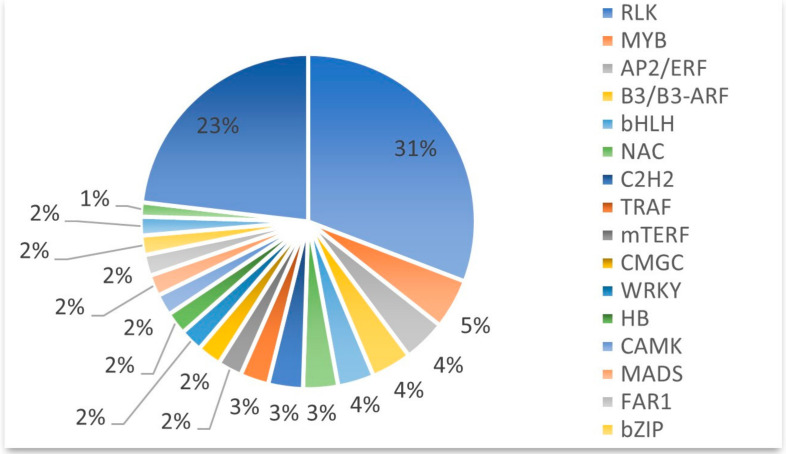
Transcription factor prediction. The percentage represents the proportion of the current transcription factor family among the total number of transcription factors.

Transcription factors that were key components involved in the transcriptional regulatory system were predicted. With the assistance of the transcription factor prediction tool (BMKCloud, www.biocloud.net (accessed on 20 March 2023)), 13,823 transcription factors with 214 types were recognized, among which we only listed the highest number of transcription factor families in the top 19 (Figure 4). The transcription factor families included transcription factors (TF, 6511 DEGs), protein kinases (PK, 5455 DEGs), and transcriptional regulators (TR, 1857 DEGs). It was simple to determine that most of the transcription factors belonged to the RLK, MYB, AP2/ERF, B3/B3-ARF, bHLH, NAC, C2H2, TRAF, mTERF, WRKY, CMGC, HB, CAMK, MADS, FAR1, bZIP, C2C2, GRAS, and other families. RLK transcription factors were most common and they were encoded by 4260 DEGs, which accounted for 31% of the total transcription factors, followed by MYB transcription factors (665 DEGs, 5%). Most of these transcription factors families play important roles in plant defense mechanisms after pathogen infection [22].

### 2.5. Candidate Resistance Gene Analysis by Integrating WGRS and RNA-Seq

**Figure 5:**
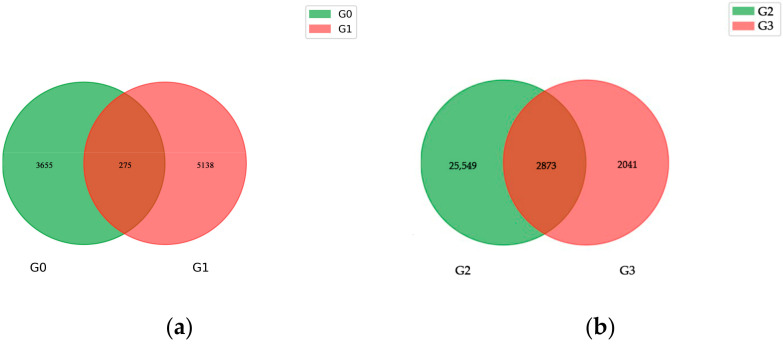

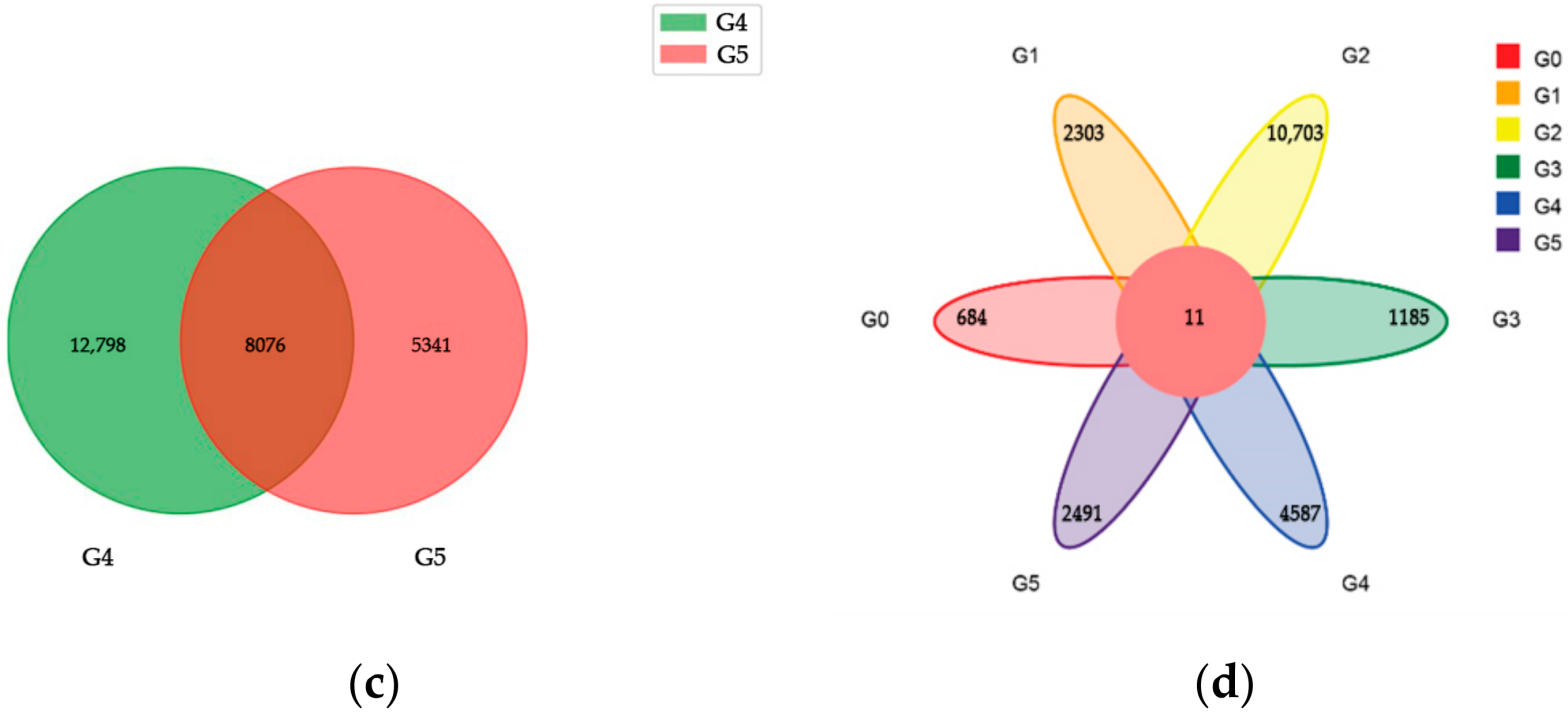
Venn diagram analysis of the DEGs in Zhongmai175 and Yili053 after TCK infection at the three growth periods. G0 represents the shared DE genes in I-YLC vs. I-YLI, G1 represents the shared DE genes in I-ZMC vs. I-ZMI, G2 represents the shared DE genes in II-YLC vs. II-YLI, G3 represents the shared DE genes in II-ZMC vs. II-ZMI, G4 represents the shared DE genes in III-YLC vs. III-YLI, and G5 represents the shared DE genes in III-ZMC vs. III-ZMI. The (**a**) represents the shared DE genes in G0 vs. G1, (**b**) represents the shared DE genes in G2 vs. G3, (**c**) represents the shared DE genes in G4 vs. G5, (**d**) represents the shared DE genes in 6 groups.

Based on a Venn diagram analysis of the DEGs in the two materials at each growth period, it was discovered that there were 275 shared genes in the mid-filling stage, 2873 shared genes in the late-filling stage, and 8076 shared genes in the maturity stage between Zhongmai175 and Yili053, which were differentially expressed in the three varieties (Figure 5a–c). Meanwhile, the shared genes in the three growth periods were examined (Figure 5d). There were eleven genes that showed consistent differential expression during the three growth periods in the two wheat varieties, and these genes were could be perceived as linked to wheat disease resistance and would be further analyzed. The distinctive DEGs and common DEGs between susceptible and resistant varieties gradually increased as the growth period changed, which may explain their differences in resistance when infected with TCK.

**Figure 6:**
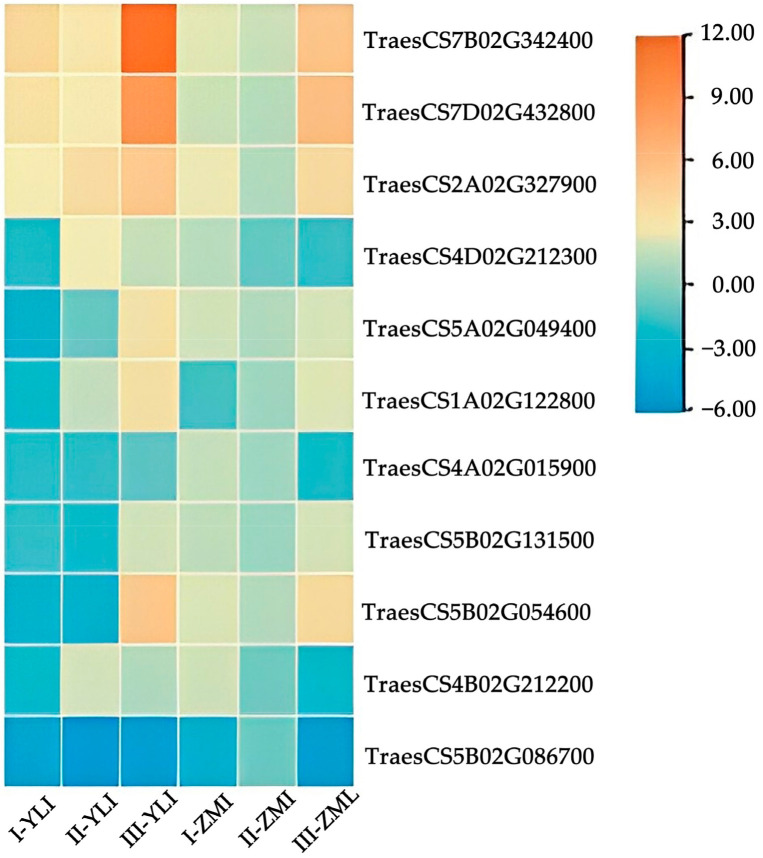
Heatmap of candidate resistant genes according to changes in expression in response to TCK infection. Orange represents positive regulation and blue represents negative regulation. The darker the color, the higher the gene expression level.

Eleven putative genes were identified that were consistently expressed over the three growth periods in the two wheat varieties. These genes were then examined if they were SNPs discovered using re-sequencing. According to a collaborative analysis, SNPs were present in nine of the eleven genes, that is, the nine genes with SNPs had transcriptional expression in wheat. The expression variations of eleven resistance genes in response to TCK infection during the three growth periods are shown in Figure 6. The expression levels of individual genes in the two materials were different. *TraesCS7B02G342400*, *TraesCS7D02G432800*, and *TraesCS2A02G327900*, three genes, were up-regulated and *TraesCS5B02G086700* was down-regulated in both varieties, other genes were either up-regulated or down-regulated in different varieties. Meanwhile, these potential TCK resistance genes are involved in key metabolic pathways, and the results are shown below (Table 3). The *TraesCS2A02G327900* gene was involved in the metabolism of tryptophan, while the other two up-regulated genes were involved in benzoxazinoid biosynthesis, and they were the genes that deserve attention.

It is a challenging task to detect and clone the quantitative traits loci (QTL) associated with disease resistance through forward genetics. Gordon [23] used a GWAS approach to identify genetic loci associated with WDB resistance. Twenty-eight accessions were highly resistant to WDB, one previously identified QTL was detected on 6DS, and they identified an additional locus on 6DS. Wang [24] identified and assessed two major QTLs for dwarf bunt resistance in winter wheat. At present, researchers have frequently used GWAS and QTL to detect and locate resistance genes related to WDB. Each method has advantages and disadvantages, and thus the combination of QTL mapping or GWAS with RNA-Seq can be used to explore disease resistance genes in a future study.

### 2.6. qRT-PCR Analysis of Candidate Resistance Genes

**Figure 7:**
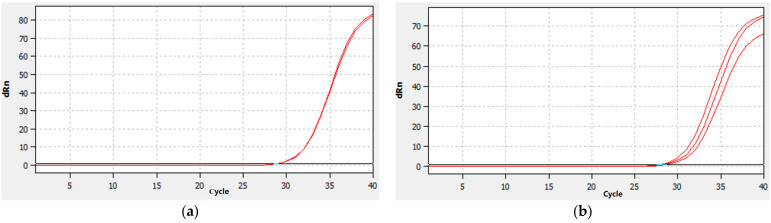
Amplification curve of DEGs that were continuously up-regulated. Note: (**a**): *TraesCS7B02G342400 gene*; (**b**): *TraesCS7D02G432800* gene. Red line represents the sample signal, the green line represents the baseline.

The 11 DEGs associated with TCK resistance were examined using qRT-PCR to determine their expression levels at 72 h after vaccination in order to confirm the validity of the RNA-sequencing data (Table 4, Figure 7). It showed that the result of RNA sequencing was accurate. The results from qRT-PCR demonstrated that the trends in the expression levels of these DEGs were consistent with those discovered using RNA sequencing, proving the validity of the transcriptome sequencing data collected in this work.

## 3. Discussion

### 3.1. Combined Analysis Based on WGRS and RNA-Seq

Wheat dwarf bunt is a type of internationally quarantined disease that could endanger China’s food production and security. The most effective method of disease control is resistant varieties, but access to disease resistance genes is extremely limited, stifling wheat breeding progress. Many plant gene sequences have been revealed as a result of the rapid development of biotechnology, which has greatly aided the advancement of plant breeding. The individual genome could be detected using WGRS technology and screened for candidate disease resistance genes based on the known reference genome sequence [25]. Meanwhile, a large number of SNPs and Indels could be obtained using WGRS [26]. Additionally, these mutants are closely related to an individual’s biological traits and are extremely important in a study screening for candidate disease resistance genes. At present, a genome wide association study (GWAS) has been used for SNP screening, but it requires a large number of samples and the sequencing cost is relatively high. Simple sequence repeat (SSR) is low in cost and has strong characteristics, but it has a low throughput. In comparison, the cost of WGRS is low, and the throughput is high. WGRS could be used to horizontally analyze the genomic differences between different varieties, creating favorable conditions for studying gene function [27]. However, the main challenge in SNP discovery is distinguishing between true biological variations and the often more abundant errors in sequence data. As a result, it is possible to reduce this error by performing a joint analysis of WGR and RNA-Seq data by observing whether SNPs are expressed during gene transcription. Wang et al. [28] analyzed the association between re-sequencing and RNA-Seq data, and found 726 and 315 InDels and SNPs that were expressed in potato, respectively. Yin et al. [29] carried out a correlation analysis of re-sequencing and RNA-Seq data in pear, and 3682 and 2067 Indels and SNPs were found to be expressed in pear. Shen [30] combined an analysis of WGRS and RNA-Seq data from kernels of two wheat cultivars, and a total of 69 840 SNPs and InDels were analyzed using transcriptome data, including 7300 genes that were expressed in wheat. In this study, in order to understand the interactions between TCK and wheat, we used WGRS and RNA-Seq technology for the first time to analyze TCK infection of wheat with varying levels of resistance. The WRGS and RNA-Seq results for identifying resistance genes in wheat were highly reliable, as evidenced by the qRT-PCR results from 11 DEGs that demonstrated the same pattern of DEG expression as established by the RNA-Seq results. Meanwhile, the key metabolic pathways of 11 genes were easier to understand the reactions of diverse wheat varieties with TCK resistance.

### 3.2. Tryptophan Metabolism and Plant Disease Resistance

Tryptophan is an important amino acid synthesized in plants. It is a crucial raw material for the production of proteins and a precursor to secondary metabolites [31]. It plays a significant role in auxin catabolism and the maintenance of auxin homeostasis in reproductive organs, which is important for plant growth and defense responses [32]. Indole glucosinolates produced from tryptophan have a vital function in plant disease and pest resistance [33]. A tryptophan derivative called phytoalexin has been demonstrated to suppress a variety of pathogens in Arabidopsis [34]. In rice, serotonin is synthesized through the branch pathway of tryptophan metabolism, which can promote the expression of defense-related genes or cell death and improved rice resistance to blast [35]. When rice was infected by *Magnaporthe oryzae*, chitin in the cell wall of pathogen was a kind of conservative pathogen-associated molecular pattern (PAMP). Rice pattern recognition receptors (PRRs) could engage with chitin directly and trigger the plant’s defense system. That is, PRRs could destroy the cell wall components of pathogens and boost the defense response of plants. The PRRs were in charge of identifying these PAMPs in plants, which belong to the receptor-like kinase (RLK) family [36]. Anthranilate synthase (AS) is an important rate-limiting enzyme for the synthesis of tryptophan in plants. Chitin had been shown to induce the expression of the gene encoding α subunit of AS, increasing AS activity and the tryptophan content, suggesting that the tryptophan biosynthesis pathway was crucial for the rice defense response [37]. In microbes, PRRs contain a variety of RLK families. In our study, 57 types of the 123 distinct protein kinases that were expressed belonged to the RLK family. However, 761 protein kinases were expressed in wheat after TCK infection, according to reference [38]. It is possible that various wheat cultivars or the difference in the calculation methods in their study caused noticeable expression discrepancies. The cell wall acted as the first barrier for pathogen contact, and the *TraesCS2A02G327900* mutation may represent a response to pathogen invasion by increasing the identification ability of TCK effectors. The mutants involved in tryptophan metabolism reduced the pathogen’s penetration by continually increasing the expression level in the two wheat varieties, which further suggested that this gene was crucial for wheat resistance to TCK infection.

### 3.3. Benzoxazinoid Biosynthesis and Plant Disease Resistance

When comparing the transcription levels of TCK-infected and healthy wheat varieties, we found that the *TraesCS7B02G342400* gene and *TraesCS7D02G432800* gene were significantly highly expressed after TCK infection. During the mid-filling stage, the expression levels in resistant varieties were significantly higher than in susceptible varieties, but at the late-filling stage and maturity stage, they were lower in susceptible varieties. According to the KEGG analysis, the two genes were mostly involved in benzoxazinoid synthesis. Benzoxazinoid is an important secondary metabolite of gramineae, which is synthesized in seedlings and stored as glucosides. Wahlroos [39] first reported that 2,4-dihydroxy-7-methoxy-2H-1,4-benzoxazosin-3(4H)-one was the predominant aglucone in wheat and maize, which had significant disease and insect resistance [40]. One of the reasons benzoxazinoid has insecticidal and antibacterial actions might be due to its ability to interact with amino acid derivatives to modify proteins [41]. The high content of benzoxazinoid in the vacuoles of the leaf cells of resistant maize cultivars greatly slowed the mycelium growth of *Setosphaeria turcics* [42]. Additionally, *Fusarium moniliforme*, *Puccinia graminis*, *Gibberella zeae*, *Cephalosporium maydis* and *Sphaerlotheca reiliana* var. Zeae were all significantly sensitive to benzoxazinoid [43]. The pathogen was first eliminated by the plant’s benzoxazinoid emission, which also killed the host cell [44]. The up-regulation of the *TraesCS7B02G342400* gene and *TraesCS7D02G432800* gene could lead to the activation of specific defense reactions, such as the modification of proteins in the cell wall, toxic secondary metabolite production, and even programmed cell death, in order to counteract TCK attack. Therefore, these two mutant genes played important roles in the response to infection with TCK. Meanwhile, the function and mechanism of benzoxazinoid derivatives, significant secondary metabolites of gramineae, could aid in the development of disease resistance breeding and provide a critical theoretical foundation for disease control.

## 4. Materials and Methods

### 4.1. Plant and Fungal Materials

The two wheat varieties were Yili 053 (sensitive variety) and Zhongmai 175 (moderately resistant variety), which were provided by the Institute of Crop Science, Chinese Academy of Agricultural Sciences, China. *Tilletia controversa* Kühn (TCK) was provided by the United States Department of Agriculture, Agricultural Research Service. The protocol in this study complied with relevant institutional, national and international guidelines and legislation.

### 4.2. Pathogen Inoculation

Wheat seeds needed to undergo vernalization treatment prior to sowing. Impurities of seeds were removed with sterile water, and seeds were soaked for eight hours. Seeds were surface-sterilized with 30% NaClO for one minute [45] and rinsed three times with sterile water. Finally, seeds covered with wet gauze at 4 °C for 30 days to vernalize. Ten vernalized seeds were planted in each pot and were grown in growth chambers (PRX-100A-LED, Tenlin, Yancheng, Jiangsu, China). Wheat seedlings were grown at 15 °C in the initial stage and at 20 °C in the maturity stage with a day/night regime of 12 h/12 h.

The OD_600_ of the teliospore suspension was 0.15 and the concentration was 10^6^ spores/mL [46]. Each treatment received 3 mL of the teliospore suspension, which was injected into the soil near the roots of seedlings five times with a one-day interval, and the control treatments received the same amount of sterile water. Three biological replicates were used for each treatment.

### 4.3. DNA Extraction, Library Construction and Whole Genome Re-Sequencing

Genomic DNA extraction from the two wheat varieties was performed according to [47]. The purity and concentration of the DNA were detected using NanoDrop 2000 (Thermo Fisher Scientific, Wilmington, DE, USA). The DNA was randomly fragmented by sonication. The DNA fragments go through terminal repair, adding A-tails and adapter ligation. The desired DNA fragments were enriched with PCR, a Truseq Nano DNA HT Sample Preparation Kit (Illumina, San Diego, CA, USA) was used to construct the sequencing library, and sequencing proceeded with the Illumina HiSeq2500 system at Biomarker Technologies Co., Ltd. (Beijing, China). Clean reads were obtained by eliminating adapter sequences and low-quality reads, mapped to the IWGSC RefSeq 1.1 reference genome (https://urgi.versailles.inra.fr/download/iwgsc/IWGSC_RefSeq_Annotations/v1.1/ (accessed on 3 February 2023)) using Burrows–Wheeler aligner (v0.7.10) [48] software with the default parameters, and the mapping output was changed to the Binary Alignment/Map (BAM) format for further analysis. Picard Tools (v1.106) [49] was used to assign BAM results and discard redundant duplicates.

### 4.4. Variant Detection and Annotation

The genomic variants were identified with the Haplotype caller module and the GVCF model from Genome analysis toolkit (GATK, v4.2.1.0) software [50]. SNPs and Indels were filtered with following criteria: QD < 2.0 || FS > 60.0 || MQ < 40.0, QD < 2.0 || FS > 200.0 || MQ < 40.0. All the variants were annotated with SnpEff (v 4.1) [51]. Regions of genomic variation (intergenic regions, gene regions, and CDS regions) and functional classes (synonymous mutations and non-synonymous mutations) were determined based on the reference genome.

### 4.5. RNA Extraction, cDNA Library Construction and Sequencing

The healthy and diseased grains from two wheat varieties were collected at the mid-filling stage, the late-filling stage and the maturity stage. Three replicates were arranged for each treatment (Table 5). Total RNA was extracted using TRNzol Universal Reagent kit (Tiangen, Beijing, China). The purity and concentration of the RNA were detected using NanoDrop 2000 (Thermo Fisher Scientific, Wilmington, DE, USA). RNA integrity was assessed using the RNA Nano 6000 Assay Kit (Agilent Technologies, Santa clara, CA, USA). The mRNA was enriched using poly-T oligo-attached magnetic beads, and were randomly interrupted in fragmentation buffer. The first strand of cDNA was synthesized using random hexamer primers and M-MuLV reverse transcriptase, while the second strand was synthesized using RNase H and the DNA polymerase I system. The cDNA fragments was purified using the AMPure XP system (Beckman Coulter, Beverly, MA, USA) in order to select cDNA fragments of preferentially 240 bp in length. The purified cDNA fragments were received by terminal repair, adding poly (A) tails and adapter ligation for PCR. The PCR products were purified using the AMPure XP system(Beckman Coulter, Beverly, MA, USA), and the quality of the library was assessed with the Agilent Bioanalyzer 2100 system. Sequencing proceeded with the Illumina HiSeq2500 system at Biomarker Technologies Co. Ltd. (Beijing, China).

### 4.6. Gene Expression and Annotation

The raw data were trimmed by eliminating adaptor sequences, sequences containing poly-N and low-quality reads from the raw data, and Q30, the GC content and clean data were calculated. The clean reads were mapped onto the IWGSC RefSeq 1.1 reference genome using the software HISAT2 (v2.0.4) [52] with the default parameters. StringTie (v1.3.3b) was used to assemble and quantify the spliced transcripts. FPKM (reads per kilobase of exon model per million mapped reads) values were used to quantify gene expression with cufflinks (v2.2.1) [53], and the differentially expressed genes (DEGs) in the two varieties at three periods were evaluated with DESeq2 [54] R package (v1.6.3), with adjusted *p* < 0.01 and logFC (fold change) > 1.5 chosen as the thresholds for significantly different expression. Candidate resistance genes were those whose differential expression was consistently observed at three stages in the two wheat varieties. Gene ontology (GO) [55], clusters of orthologous groups terms (COG) [56] and Kyoto Encyclopedia of Genes and Genomes (KEGG) [57] enrichment analyses were conducted for DEGs with the clusterProfiler R package (v3.10.1), and *p*-values < 0.05 were considered significantly enriched DEGs.

### 4.7. Expression Profiles of Candidate Resistance Genes Using qRT-PCR

qRT-PCR was used to confirm the expression levels of candidate resistance genes. The extraction of total RNA and library construction for 11 candidate resistance genes were identical to the previous methods. The GAPDH gene was used as an endogenous control. The primers used in this experiment are listed in Table 6. Three replicates were used for each gene. Following the manufacturer’s instructions, qRT-PCR was performed in a volume of 20 L using Top Green qPCR SuperMix (TransGen, Beijing, China) and applied to the QuantStudio 5 instrument (Applied Biosystems, Beijing, China). All genes were subjected to pre-denaturation at 95 °C for 10 min, followed by 40 cycles (95 °C for 15 s (denaturation), 58 °C for 30 s (extension), and 72 °C for 30 s). The specificity of the amplification was verified through a melting curve analysis (from 60 to 95 °C). The expression level of each gene was calculated using the 2^−ΔΔCt^ method [58].

## 5. Conclusions

This study was the first to use whole genome re-sequencing and RNA sequencing to investigate putative TCK resistance genes in wheat by comparing resistant and susceptible wheat. Eleven DEGs associated with disease resistance were discovered using a transcriptome analysis, and SNP variations in genes in the two wheat types were discovered using re-sequencing. Nine candidate resistance genes were found to have SNP mutations through the combined analysis of RNA sequencing and re-sequencing data, and three of them displayed continuous up-regulation, which were predicted to be related to plant disease resistance. One gene was predicted to be related to tryptophan metabolism; two genes were related to the synthesis of benzoxazinoid. These findings served as a guide for comprehending the genetic variance between Zhongmai 175 and Yili 053, as well as the likely mechanism of resistance to TCK infection. They could also serve as a theoretical foundation for breeding disease-resistant plants.

## Figures and Tables

**Table 1:** Number of variations in the two wheat samples.

Variety	Number of SNPs	Numberof InDels	Genes with Non-Synonymous SNPs	Genes with InDels	Total Genes with Variations
Zhongmai 175	24,085,517	2,203,974	30,742	9625	32,417
Yili 053	25,534,555	2,318,474	31,512	9647	33,171

**Table 2:** Statistical functional annotation of DEGs.

Group	DEG Set	Total	GO	KEGG	COG
Group 1	I-YLC vs. I-YLI	3683	2941	2406	1383
Group 2	I-ZMC vs. I-ZMI	15,525	11,858	10,029	5306
Group 3	II-YLC vs. II-YLI	1117	730	606	303
Group 4	II-ZMC vs. II-ZMI	4691	3688	3119	1799
Group 5	III-YLC vs. III-YLI	24,877	19,451	16,697	8934
Group 6	III-ZMC vs. III-ZMI	10,535	7992	6868	3668

**Table 3:** Functional annotation of candidate resistance genes.

Gene ID	Regulation						KEGG Annotation	SNP Mutation
	I-YLC vs. I-YLI	II-YLC vs. II-YLI	III-YLC vs. III-YLI	I-ZMC vs. I-ZMI	II-ZMC vs. II-ZMI	III-ZMC vs. III-ZMI		
*TraesCS7B02G342400*	up	up	up	up	up	up	Benzoxazinoid biosynthesis (ko00402)	Yes
*TraesCS7D02G432800*	up	up	up	up	up	up	Benzoxazinoid biosynthesis (ko00402)	Yes
*TraesCS2A02G327900*	up	up	up	up	up	up	Tryptophan metabolism (ko00380)	Yes
*TraesCS5A02G049400*	down	down	up	up	up	up	Protein processing in endoplasmic reticulum (ko04141)	No
*TraesCS5B02G131500*	down	down	up	up	up	up	--	Yes
*TraesCS5B02G054600*	down	down	up	up	up	up	Protein processing in endoplasmic reticulum (ko04141)	Yes
*TraesCS5B02G086700*	down	down	down	down	down	down	--	Yes
*TraesCS4A02G015900*	down	down	down	up	up	down	Alanine, aspartate and glutamate metabolism (ko00250)	Yes
*TraesCS4B02G212200*	down	up	up	up	down	down	Glycine, serine and threonine metabolism (ko00260)	No
*TraesCS4D02G212300*	down	up	up	up	down	down	Cysteine and methionine metabolism (ko00270)	Yes
*TraesCS1A02G122800*	down	up	up	down	up	up	Valine, leucine and isoleucine degradation (ko00280)	Yes

**Table 4:** Validation of RNA-Seq data using qRT-PCR.

Gene ID	ZM175			YL053		
	qRT-PCR	FPKM	Validated	qRT-PCR	FPKM	Validated
*TraesCS7B02G342400*	0.15 ± 0.21 c	110.78	+	0.07 ± 0.06 ab	175.8	+
*TraesCS7D02G432800*	0.38 ± 0.06 bc	78.11	+	0.61 ± 0.31 ab	79.31	+
*TraesCS2A02G327900*	0.31 ± 0.01 c	24.2	+	0.31 ± 0.17 ab	52.97	+
*TraesCS5A02G049400*	0.85 ± 0.1 bc	11.8	+	0.78 ± 0.19 ab	39.71	+
*TraesCS5B02G131500*	1.29 ± 0.11 bc	241.8	+	1.1 ± 1.57 ab	235.09	+
*TraesCS5B02G054600*	1.03 ± 1.07 bc	15.11	+	0.56 ± 0.45 ab	15.42	+
*TraesCS5B02G086700*	2.06 ± 0.03 ab	10.41	+	0.22 ± 0.07 ab	8.78	+
*TraesCS4A02G015900*	0.72 ± 0.42 bc	30.87	+	1.47 ± 0.98 ab	48.21	+
*TraesCS4B02G212200*	3.27 ± 2.46 a	111.19	+	2.16 ± 2.39 ab	329.83	+
*TraesCS4D02G212300*	1.14 ± 0.68 bc	38.7	+	2.33 ± 1.85 a	147.36	+
*TraesCS1A02G122800*	0.34 ± 0.02 c	11.89	+	0.23 ± 0.14 ab	12.52	+

Note: a–c represents significant difference in the amount of gene expression detected by qRT-PCR.

**Table 5:** Sampling and nomenclature.

Sample Collection Time	Character	Name of Zhongmai 175	Name of Yili 053
Mid-filling stage	Diseased	Ⅰ-ZMI1	Ⅰ-YLI1
		Ⅰ-ZMI2	Ⅰ-YLI2
		Ⅰ-ZMI3	Ⅰ-YLI3
	Healthy	Ⅰ-ZMC1	Ⅰ-YLC1
		Ⅰ-ZMC2	Ⅰ-YLC2
		Ⅰ-ZMC3	Ⅰ-YLC3
Late-filling stage	Diseased	Ⅱ-ZMI1	Ⅱ-YLI1
		Ⅱ-ZMI2	Ⅱ-YLI2
		Ⅱ-ZMI3	Ⅱ-YLI3
	Healthy	Ⅱ-ZMC1	Ⅱ-YLC1
		Ⅱ-ZMC2	Ⅱ-YLC2
		Ⅱ-ZMC3	Ⅱ-YLC3
Maturity stage	Diseased	Ⅲ-ZMI1	Ⅲ-YLI1
		Ⅲ-ZMI2	Ⅲ-YLI2
		Ⅲ-ZMI3	Ⅲ-YLI3
	Healthy	Ⅲ-ZMC1	Ⅲ-YLC1
		Ⅲ-ZMC2	Ⅲ-YLC2
		Ⅲ-ZMC3	Ⅲ-YLC3

**Table 6:** Primers for selected DEGs in the expression analysis using qRT-PCR.

Gene ID	Forward Primer (5′-3′)	Reverse Primer (5′-3′)
*TraesCS7B02G342400*	GTCCAACGACAGGTTCCG	CACACCGTAGCTCCCTTG
*TraesCS7D02G432800*	TTCCTCACCGTGCTGCTC	GTTCCCTTCGCCGTCCTC
*TraesCS2A02G327900*	GGAGACCGTCAAGAGCTA	GTGTCCTGCGTGTAGTTG
*TraesCS5A02G049400*	CAGCATCTGCCTGGAGAC	GACTTGGACCTGGAGGAG
*TraesCS5B02G131500*	GACCTGCTGAAAGGATCT	TTGTTCTTGGGATTCTCGTA
*TraesCS5B02G054600*	ATGACATCTTCGGAGTACC	GACTTGGACTTGGAGGAG
*TraesCS5B02G086700*	CATCCGAAGCAGCAGTCT	GCTTGGAGACGATGGACT
*TraesCS4A02G015900*	GAATCGGCAACGGTCTAC	CCAATCCACCAGCAGAAC
*TraesCS4B02G212200*	CTGAAGAAGGAGGAGGTG	AGGAACTTACCACTGCTG
*TraesCS4D02G212300*	AAGAAGGAGCAGGAGGAG	TCTGGATGGACTTGACCT
*TraesCS1A02G122800*	ACATTAAACGCACCAACCT	ACCATCTTCCGAGTCTCC

## Data Availability

Data are contained within the article.

## Supplementary Materials

The supporting information can be downloaded at https://www.mdpi.com/article/10.3390/ijms242417356/s1.
