# Supplementary material for: Combining Transcriptome and Whole Genome Re-Sequencing to Screen Disease Resistance Genes for Wheat Dwarf Bunt
Source: Int J Mol Sci. 2023 Dec 11;24(24):17356. doi: 10.3390/ijms242417356 (PMC10743994; doi:10.3390/ijms242417356)
Supplement: Supplementary file 1 [file ijms-24-17356-s001.zip › ijms-2662828-supplementary.pdf]

**Table S1 Sample sequencing data statistics**

| Varieties | Clean_Reads | Clean_Base | Q30(%) | GC(%) |
|---|---|---|---|---|
| Zhongmai 175 | 1 013 423 192 | 303 651 869 238 | 93.61 | 46.46 |
| Yili 053 | 1 098 471 243 | 329 150 128 306 | 93.72 | 47.66 |

**Table S2 Statistical results of comparison between samples and reference genomes**

| Varieties | Total_reads | Mapped(%) | Properly_mapped(%) |
|---|---|---|---|
| Zhongmai 175 | 2 026 846 384 | 99.37 | 96.18 |
| Yili 053 | 2 196 942 486 | 99.67 | 96.33 |

**Table S3 Statistical results of detected SNPS**

| Varieties | SNPnumber | Transition | Transversion | Ti/Tv | Heterozygosity | Homozygosity | Het-ratio |
|---|---|---|---|---|---|---|---|
| Zhongmai 175 | 24085517 | 17103510 | 6982007 | 2.44 | 3927004 | 20158513 | 16.3% |
| Yili 053 | 25534555 | 18137285 | 7397270 | 2.45 | 4042195 | 21492360 | 15.83% |

**Table S4 The distribution of the InDel**

| Varieties | CDS-Insertion | CDS-Deletion | CDS-Homo | CDS-Het | CDS-Total | Ratio of Insertion | Ratio of Deletion |
|---|---|---|---|---|---|---|---|
| Zhongmai 175 | 7154 | 7925 | 7313 | 7766 | 15079 | 47.44% | 52.56% |
| Yili 053 | 7012 | 7680 | 7389 | 7303 | 14692 | 47.73% | 52.27% |
| Total | 11050 | 12200 | -- | -- | 23250 | - | - |
| | Genome-Insertion | Genome-Deletion | Genome-Homo | Genome-Het | Genome-Total | Ratio of Insertion | Ratio of Deletion |
| Zhongmai 175 | 1059702 | 1144272 | 1710302 | 493672 | 2203974 | 48.08% | 51.92% |
| Yili 053 | 1119222 | 1199252 | 1811166 | 507308 | 2318474 | 48.27% | 51.73% |
| Total | 1566939 | 1707589 | -- | -- | 3274528 | - | - |

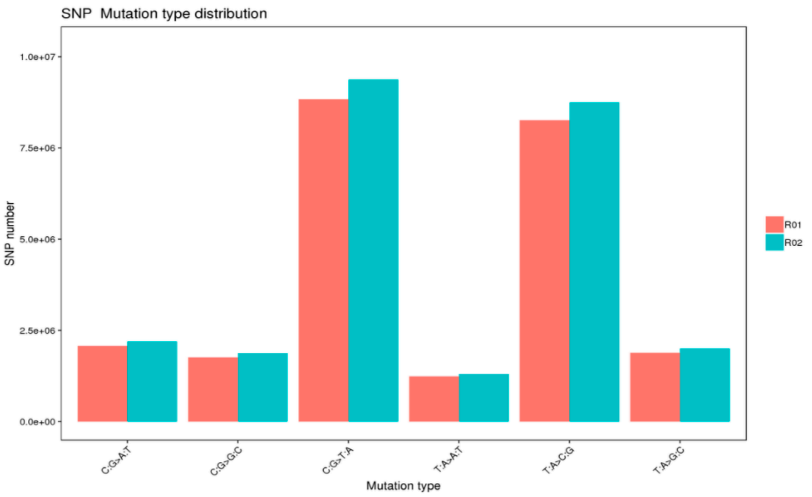


**Figure S1 Distribution of the SNP mutation type**

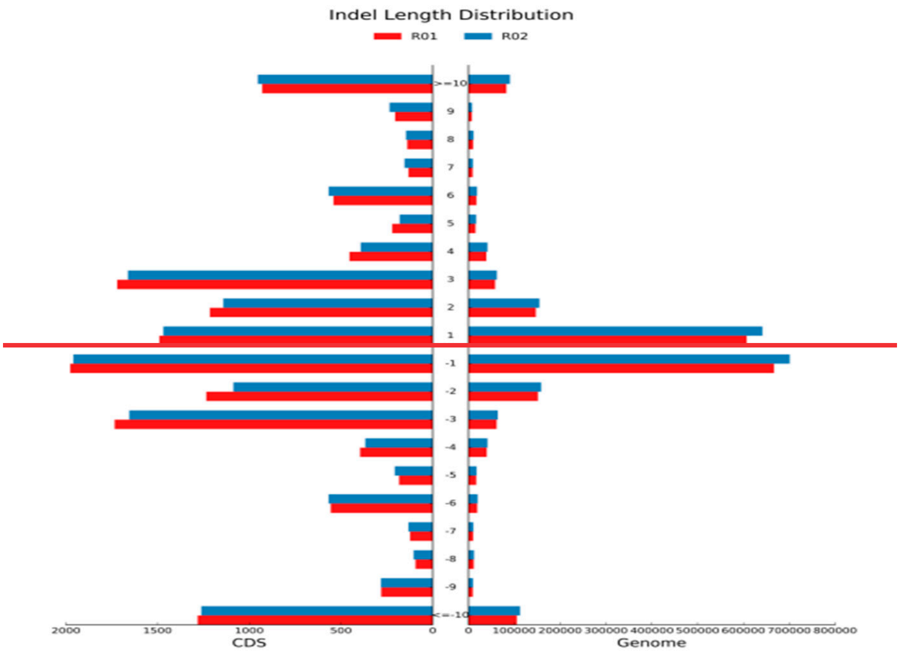
Indel Length Distribution
R01
R02
>=10
9
8
7
6
5
4
3
2
1
-1
-2
-3
-4
-5
-6
-7
-8
-9
<=-10
2000 1500 1000 500 0
0 100000 200000 300000 400000 500000 600000 700000 800000
CDS
Genome

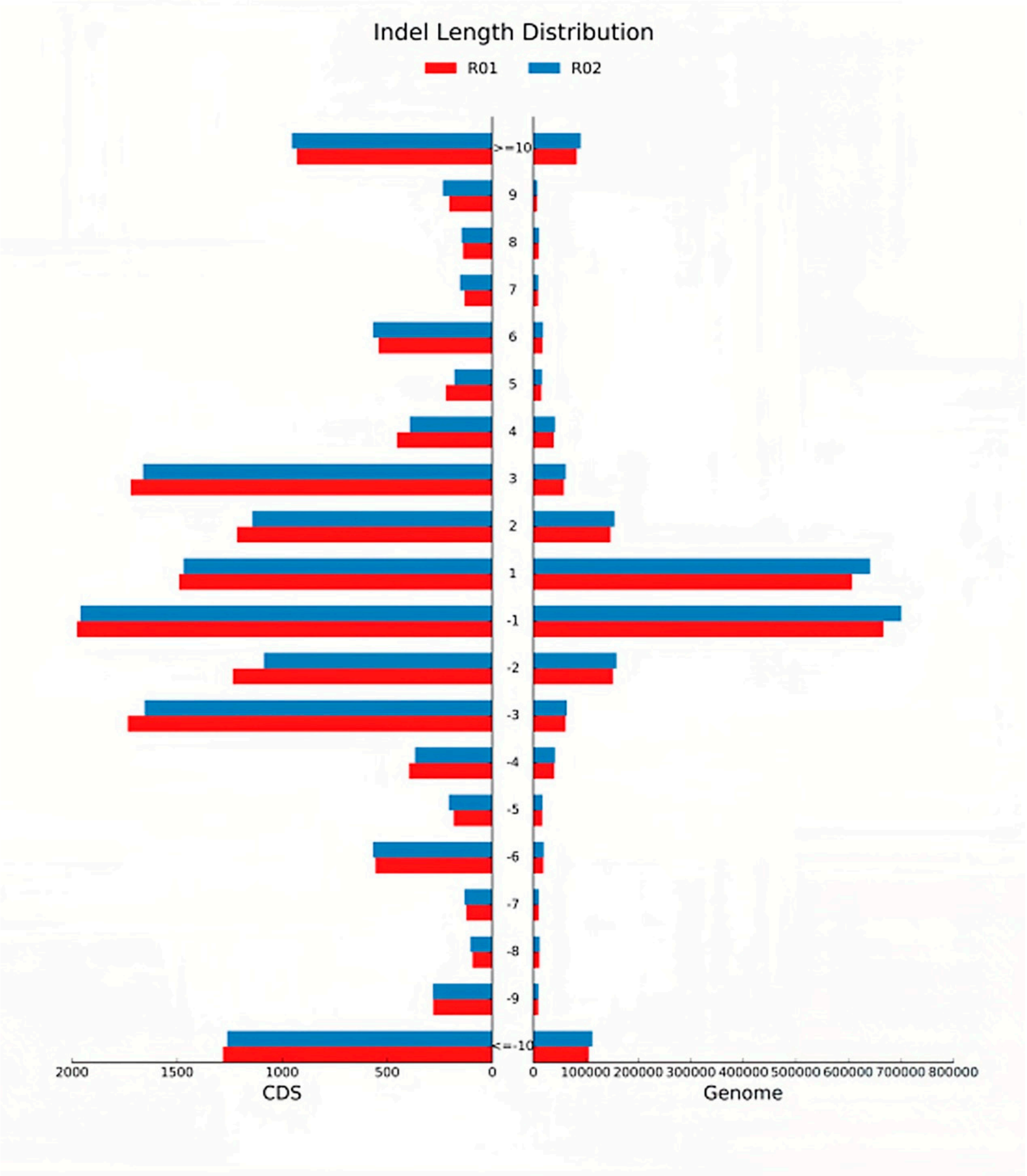


**Figure S2 Distribution of the InDels type**

**Note: The ordinate is the InDel length (within 10bp), greater than 0 is Insertion, less than 0 is Deletion, and the abscissa is the corresponding quantity.**

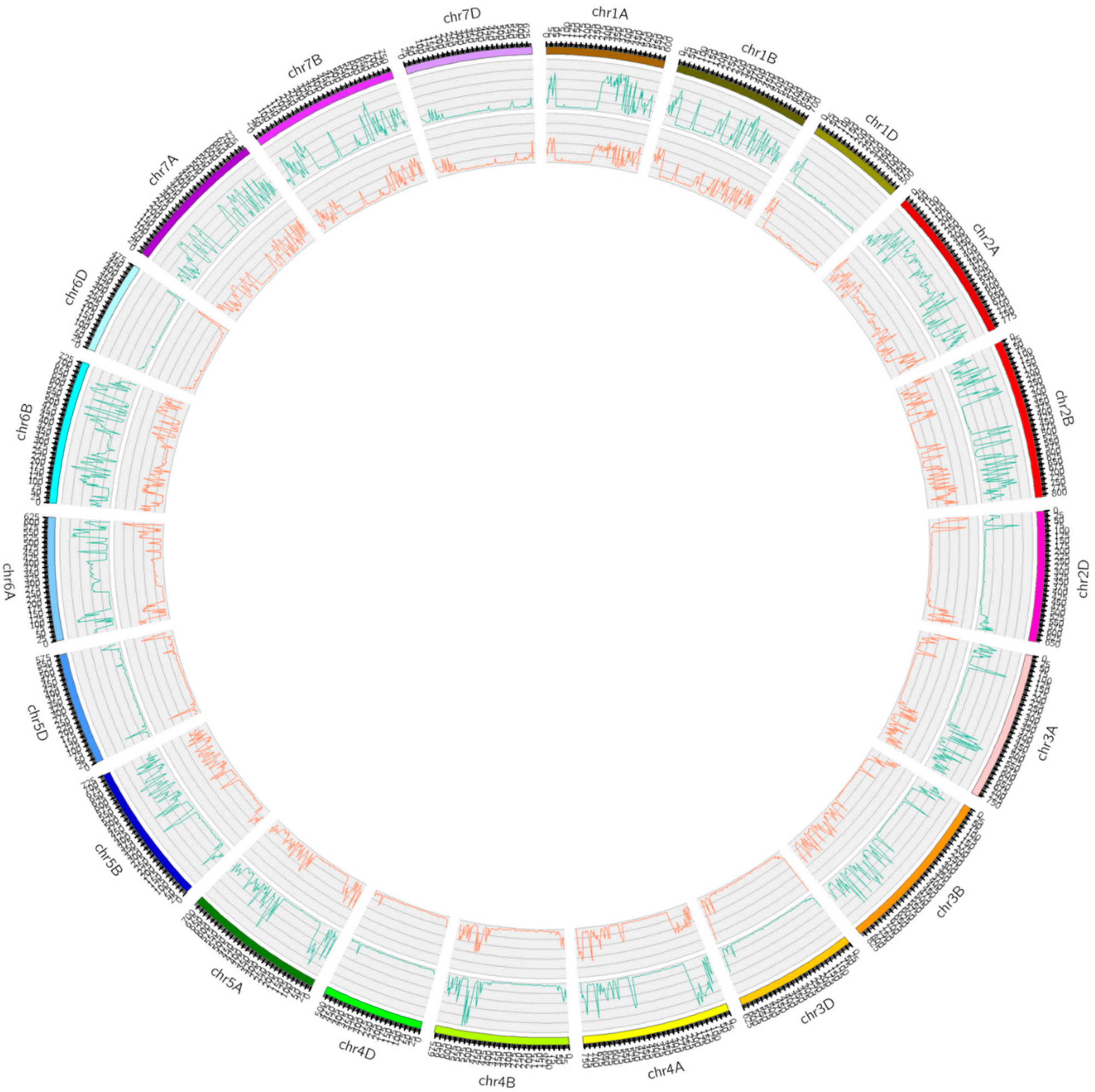
chr1A
chr1B
chr1D
chr2A
chr2B
chr2D
chr3A
chr3B
chr3D
chr4A
chr4B
chr4D
chr5A
chr5B
chr5D
chr6A
chr6B
chr6D
chr7A
chr7B
chr7D

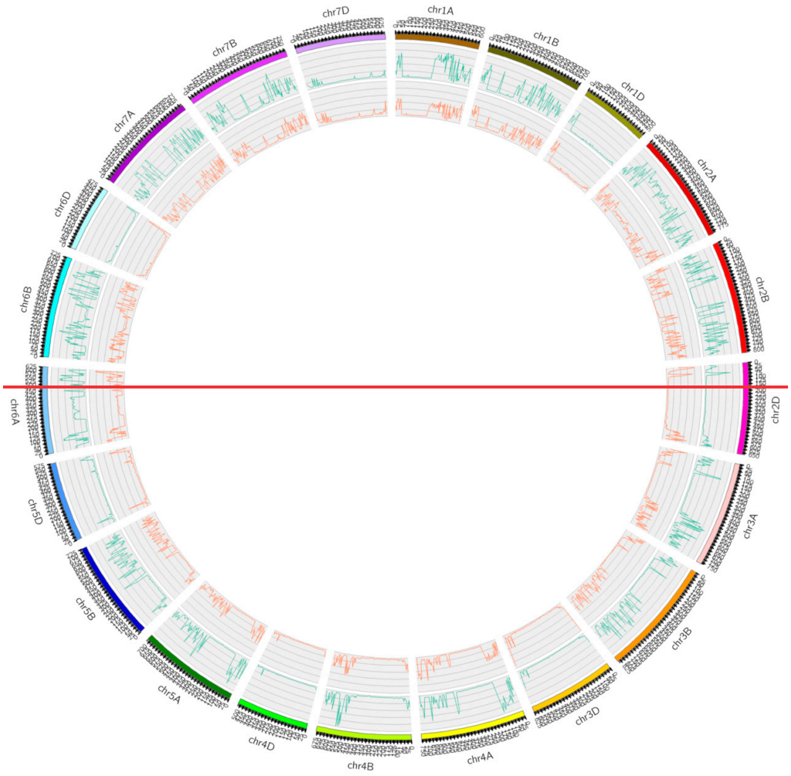
chr1A
chr1B
chr1D
chr2A
chr2B
chr2D
chr3A
chr3B
chr3D
chr4A
chr4B
chr4D
chr5A
chr5B
chr5D
chr6A
chr6B
chr6D
chr7A
chr7B
chr7D

**(a) SNP**

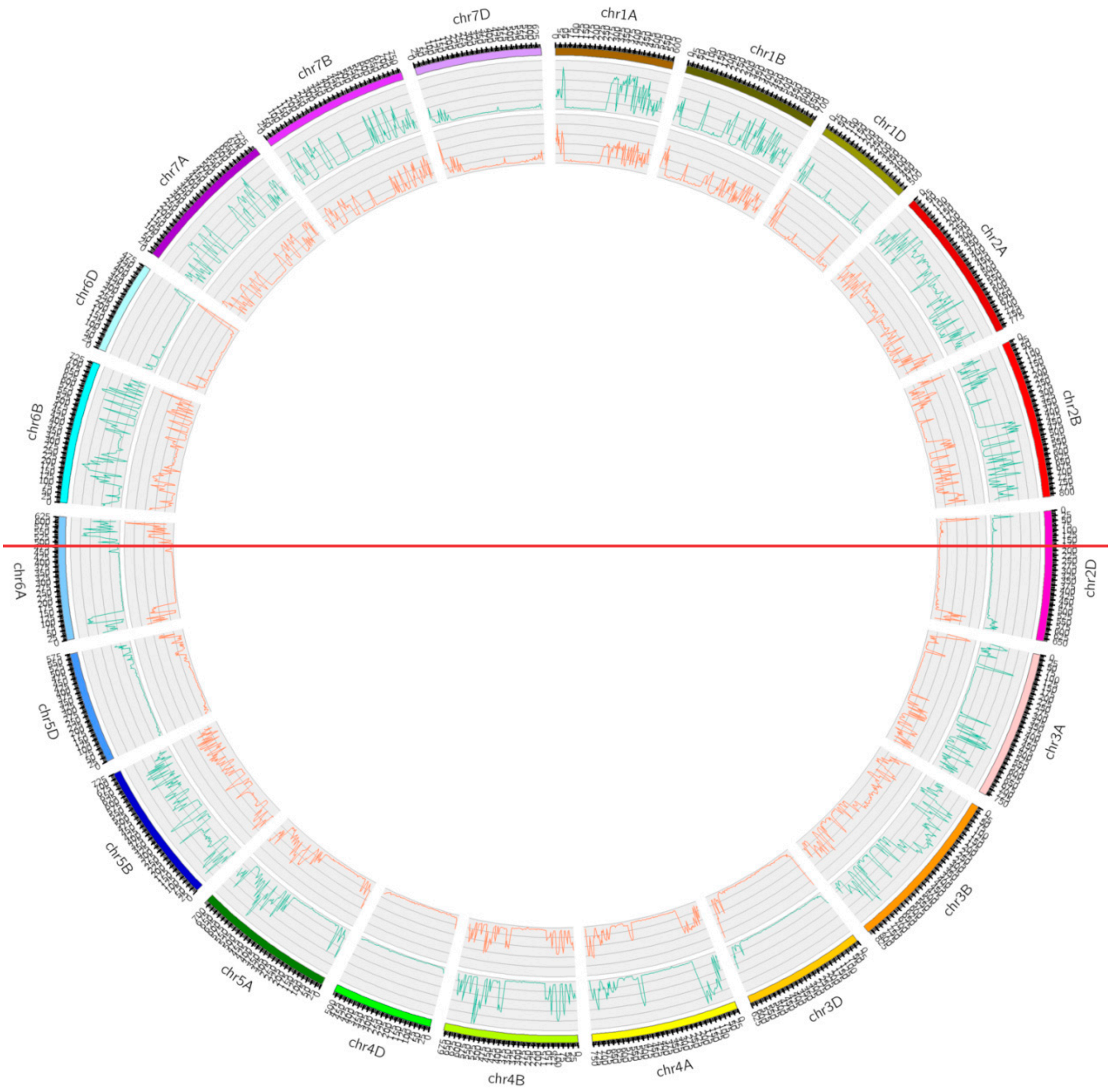
chr1A
chr1B
chr1D
chr2A
chr2B
chr2D
chr3A
chr3B
chr3D
chr4A
chr4B
chr4D
chr5A
chr5B
chr5D
chr6A
chr6B
chr6D
chr7A
chr7B
chr7D

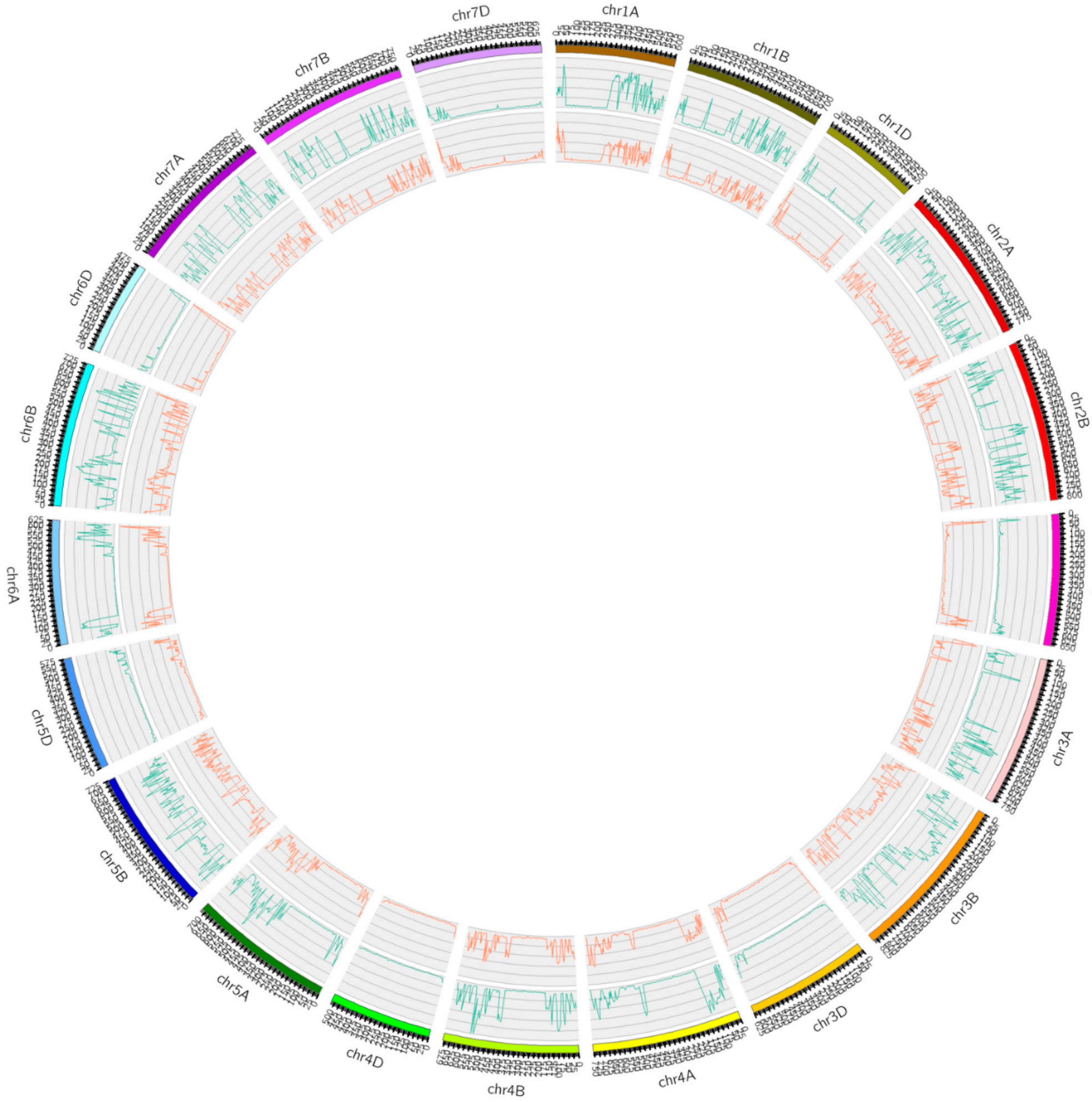


**(b) InDel**

**Figure S3 From outside to inside, the distribution of chromosome coordinates, SNP density distribution and InDel density distribution on the genome (unit: M).**

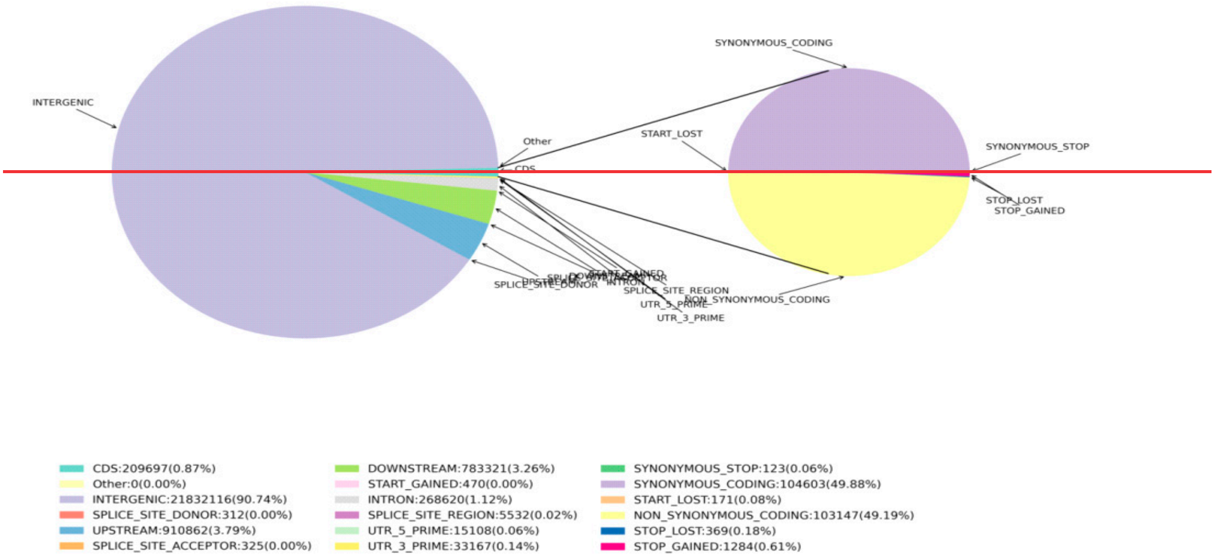


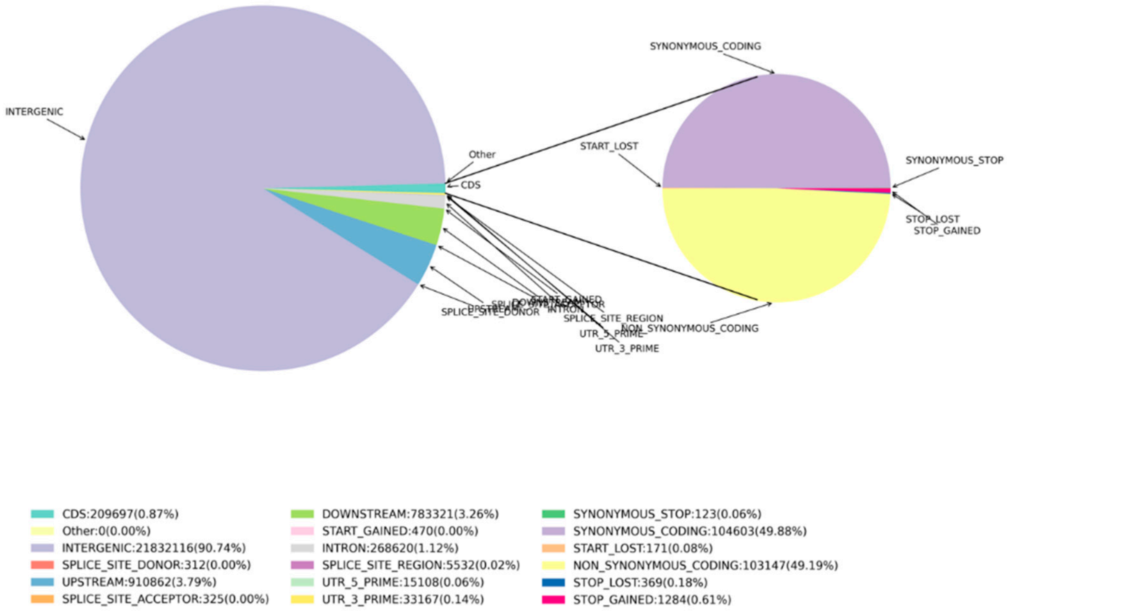


**(a) Zhongmai175-SNPs**

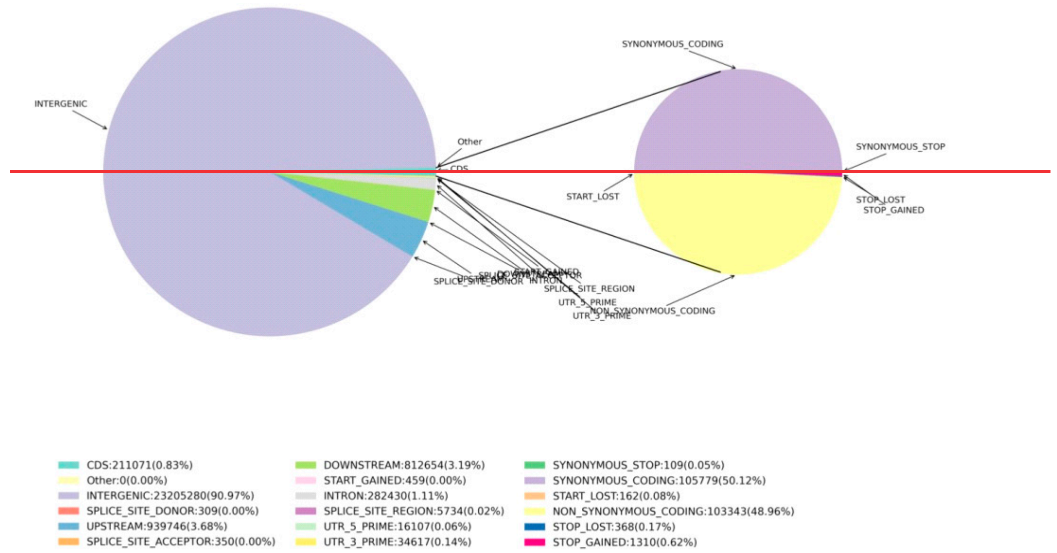


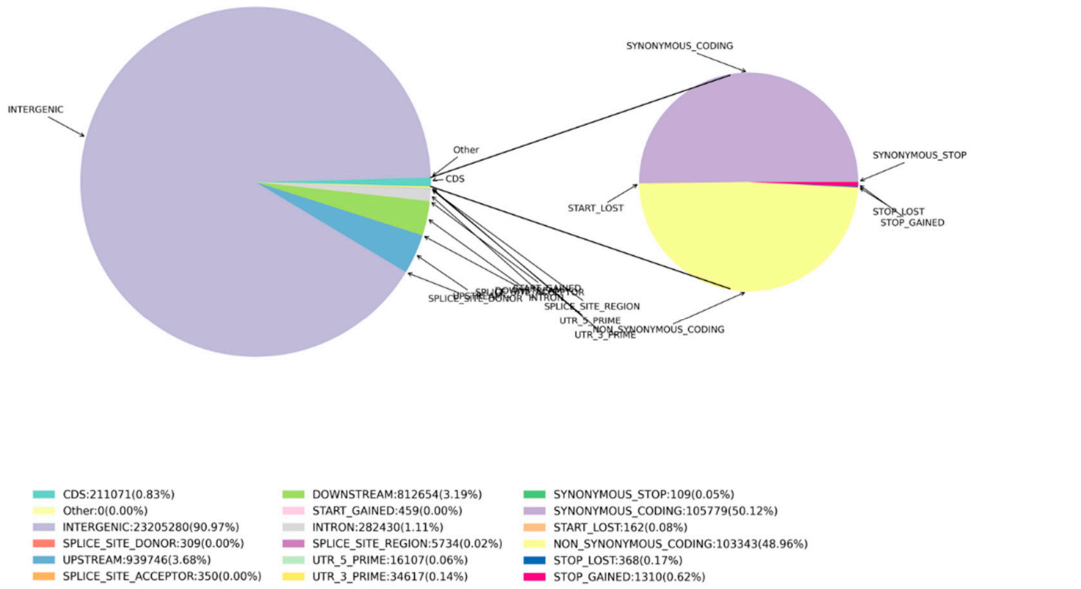


**(b) Yili35-SNPs**

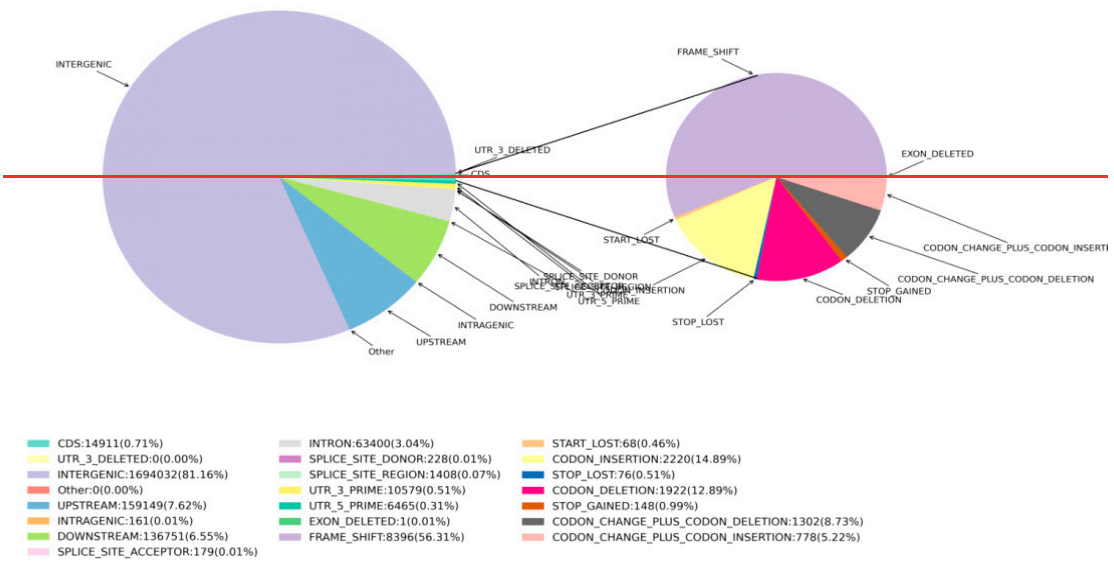


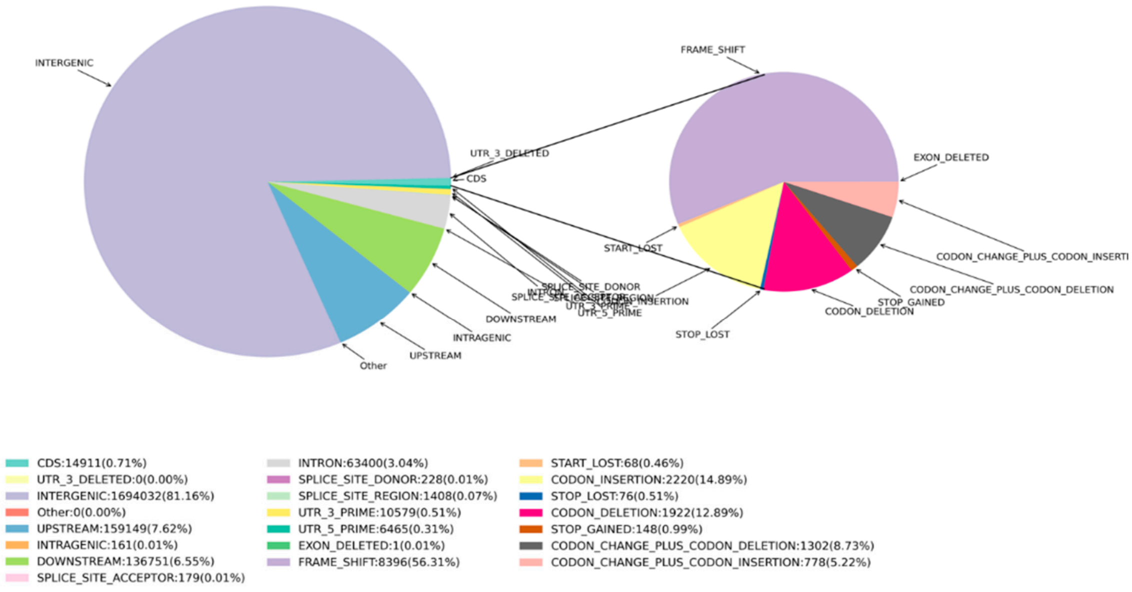


**(c) Zhongmai175- InDels**

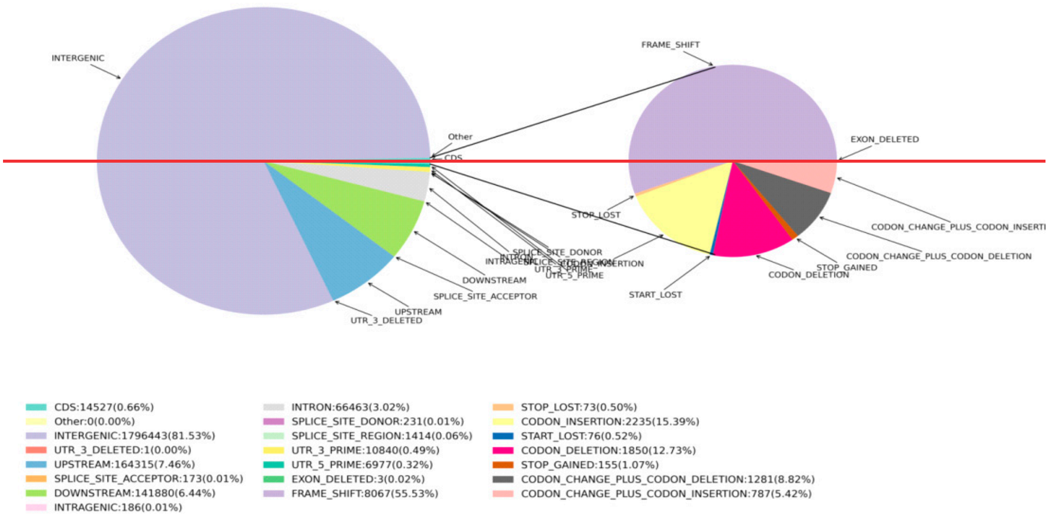


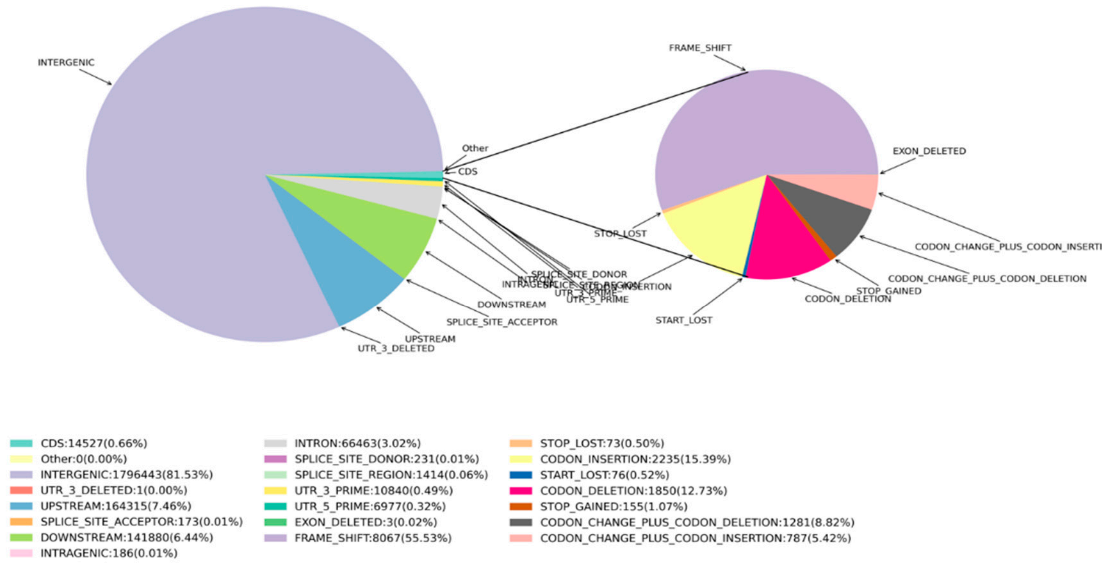


**(d) Yili35-InDels**

**Figure S4 The distribution of detected SNPs and InDels**

**Table S5 Statistical of RNA sequencing data**

| Name | Clean reads | Clean bases | GC Content (%) | %≥Q30 (%) |
|---|---|---|---|---|
| I-YLC1 | 33 846 470 | 10 118 821 246 | 55.69 | 95.46 |

| | | | | |
|---|---|---|---|---|
| I-YLC2 | 38 588 627 | 11 517 825 572 | 54.84 | 95.00 |
| I-YLC3 | 37 958 897 | 11 343 101 536 | 54.37 | 95.01 |
| I-YLI1 | 37 731 986 | 11 276 627 552 | 57.82 | 93.91 |
| I-YLI2 | 35 408 404 | 10 561 674 364 | 58.70 | 93.97 |
| I-YLI3 | 43 665 455 | 13 042 812 834 | 58.55 | 93.90 |
| I-ZMC1 | 46 351 695 | 13 859 971 274 | 53.30 | 93.61 |
| I-ZMC2 | 33 043 832 | 9 877 042 464 | 53.23 | 93.59 |
| I-ZMC3 | 39 213 222 | 11 719 342 324 | 53.53 | 93.86 |
| I-ZMI1 | 39 334 112 | 11 736 793 442 | 57.90 | 94.08 |
| I-ZMI2 | 34 939 631 | 10 434 142 052 | 58.39 | 94.07 |
| I-ZMI3 | 42 808 935 | 12 810 248 222 | 58.27 | 93.61 |
| II-YLC1 | 37 424 109 | 11 172 643 120 | 51.80 | 91.54 |
| II-YLC2 | 39 668 019 | 11 817 094 732 | 52.53 | 91.17 |
| II-YLC3 | 43 770 044 | 13 052 803 514 | 51.85 | 91.92 |
| II-YLI1 | 41 044 118 | 12 272 430 466 | 56.42 | 93.91 |
| II-YLI2 | 41 626 954 | 12 447 001 058 | 56.32 | 93.99 |
| II-YLI3 | 43 680 494 | 13 053 542 618 | 56.40 | 93.96 |
| II-ZMC1 | 43 848 261 | 13 091 674 164 | 52.32 | 90.85 |
| II-ZMC2 | 46 930 995 | 14 010 256 938 | 52.47 | 91.39 |
| II-ZMC3 | 35 503 431 | 10 596 875 466 | 52.88 | 91.04 |
| II-ZMI1 | 32 780 524 | 9 760 655 060 | 52.15 | 90.77 |
| II-ZMI2 | 40 222 234 | 11 979 004 022 | 52.35 | 90.79 |
| II-ZMI3 | 44 797 780 | 13 334 563 452 | 52.26 | 90.97 |
| III-YLC1 | 41 024 047 | 12 249 838 096 | 57.04 | 93.24 |
| III-YLC2 | 42 236 376 | 12 634 731 488 | 56.04 | 94.14 |
| III-YLC3 | 36 526 906 | 10 892 077 864 | 56.40 | 93.28 |
| III-YLI1 | 43 832 299 | 13 101 853 390 | 58.51 | 93.54 |
| III-YLI2 | 43 363 058 | 12 976 495 304 | 58.83 | 93.85 |
| III-YLI3 | 45 101 790 | 13 488 891 936 | 58.73 | 93.80 |
| III-ZMC1 | 37 741 594 | 11 253 577 368 | 55.98 | 93.36 |
| III-ZMC2 | 36 194 533 | 10 817 335 954 | 56.65 | 93.34 |
| III-ZMC3 | 37 219 406 | 11 087 564 962 | 55.67 | 93.42 |
| III-ZMI1 | 32 791 493 | 9 791 366 044 | 58.57 | 93.87 |
| III-ZMI2 | 36 361 003 | 10 873 095 430 | 58.61 | 94.10 |
| III-ZMI3 | 33 880 154 | 10 127 859 878 | 58.35 | 93.70 |

**Table S6 Statistical of DEG in two varieties at different growth period**

| DEG Set | DEG Number | up-regulated | down-regulated |
|---|---|---|---|
| I-YLC1_I-YLC2_I-YLC3_vs_I-YLI1_I-YLI2_I-YLI3 | 3,930 | 1,148 | 2,782 |

| | | | |
|---|---|---|---|
| I-YLC1_I-YLC2_I-YLC3_vs_I-ZMC1_I-ZMC2_I-ZMC3 | 19,128 | 13,312 | 5,816 |
| I-YLI1_I-YLI2_I-YLI3_vs_I-ZMI1_I-ZMI2_I-ZMI3 | 1,604 | 1,377 | 227 |
| I-ZMC1_I-ZMC2_I-ZMC3_vs_I-ZMI1_I-ZMI2_I-ZMI3 | 5,413 | 2,710 | 2,703 |
| II-YLC1_II-YLC2_II-YLC3_vs_II-YLI1_II-YLI2_II-YLI3 | 28,422 | 11,405 | 17,017 |
| II-YLC1_II-YLC2_II-YLC3_vs_II-ZMC1_II-ZMC2_II-ZMC3 | 12,720 | 6,077 | 6,643 |
| II-YLI1_II-YLI2_II-YLI3_vs_II-ZMI1_II-ZMI2_II-ZMI3 | 30,114 | 17,406 | 12,708 |
| II-ZMC1_II-ZMC2_II-ZMC3_vs_II-ZMI1_II-ZMI2_II-ZMI3 | 4,914 | 2,984 | 1,930 |
| III-YLC1_III-YLC2_III-YLC3_vs_III-YLI1_III-YLI2_III-YLI3 | 20,874 | 7,374 | 13,500 |
| III-YLC1_III-YLC2_III-YLC3_vs_III-ZMC1_III-ZMC2_III-ZMC3 | 7,188 | 3,473 | 3,715 |
| III-YLI1_III-YLI2_III-YLI3_vs_III-ZMI1_III-ZMI2_III-ZMI3 | 1,183 | 197 | 986 |
| III-ZMC1_III-ZMC2_III-ZMC3_vs_III-ZMI1_III-ZMI2_III-ZMI3 | 13,417 | 1,752 | 11,665 |

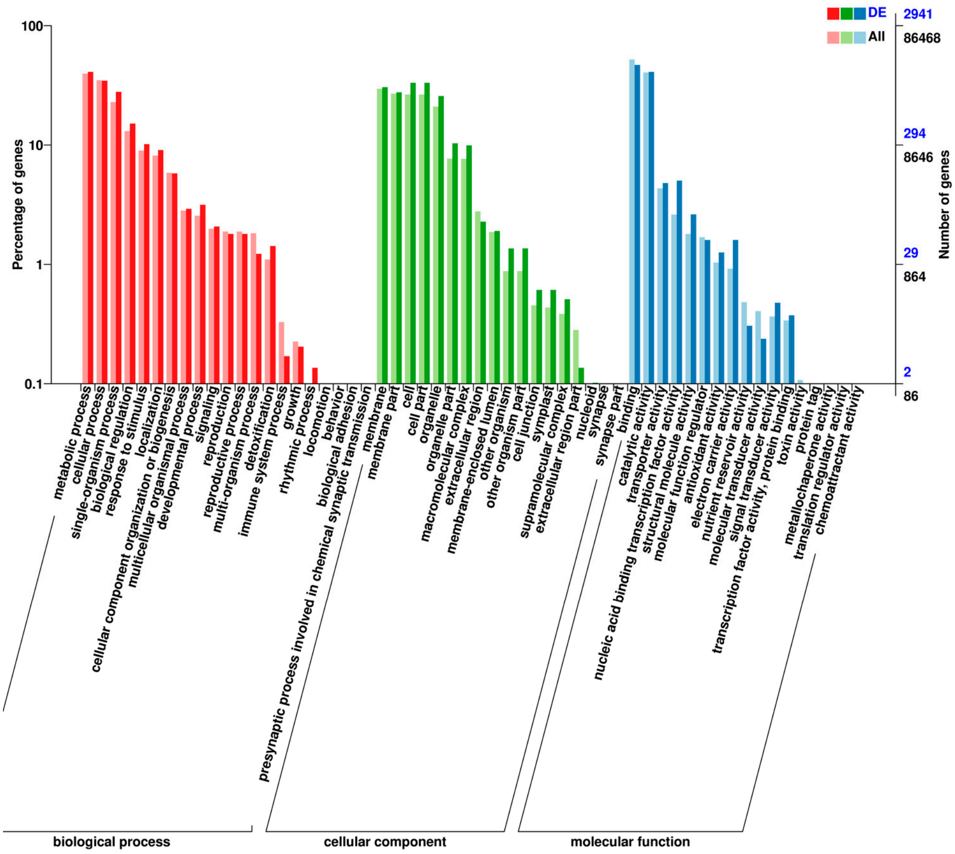


group 1: I-YLC vs I-YLI;

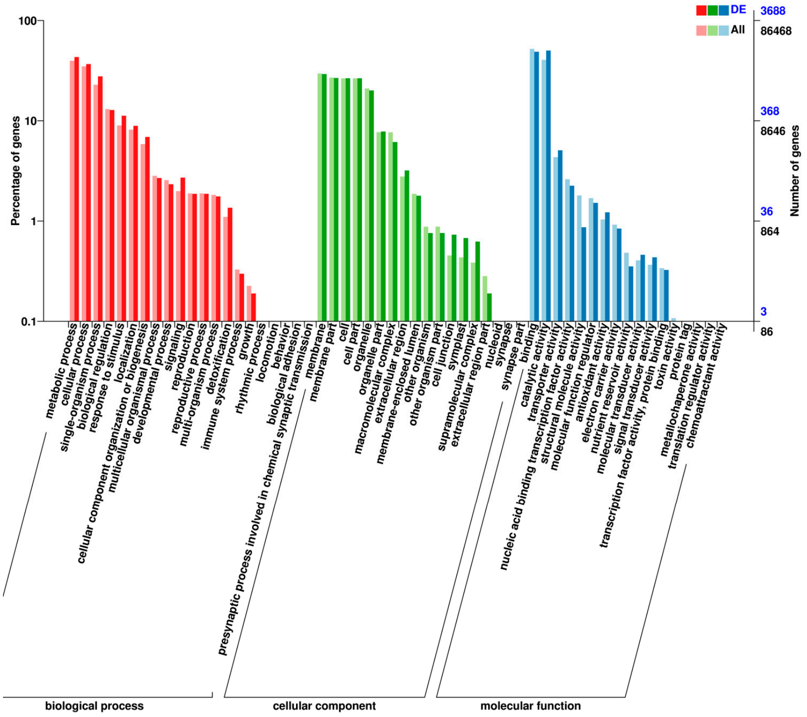


group2: I-ZMC vs I-ZMI;

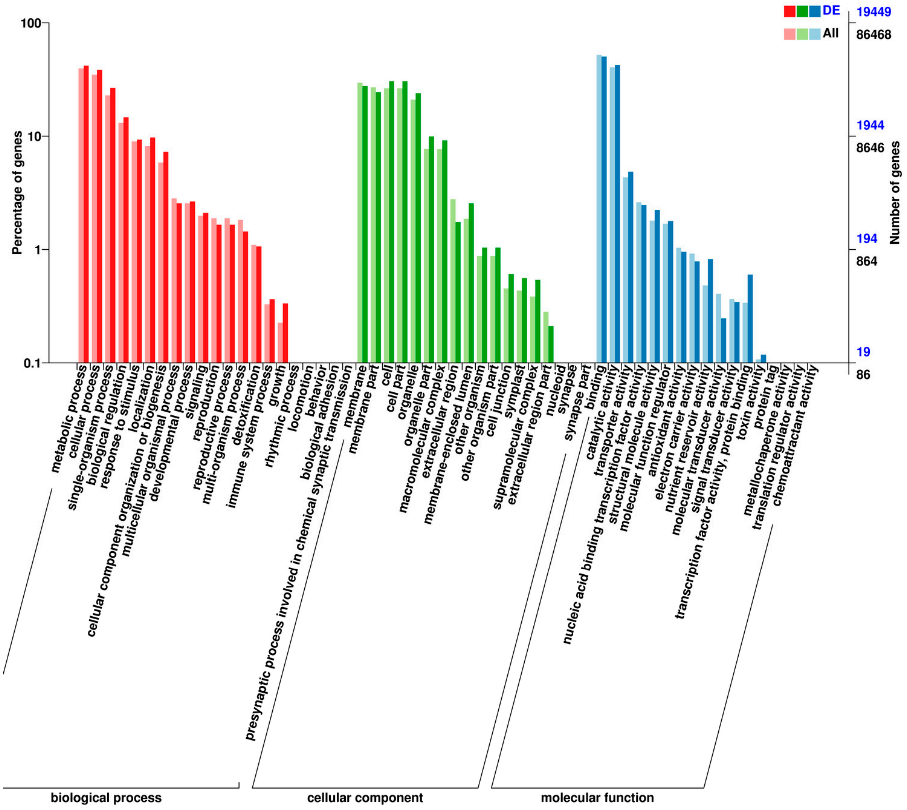


group3: II-YLC vs II-YLI;

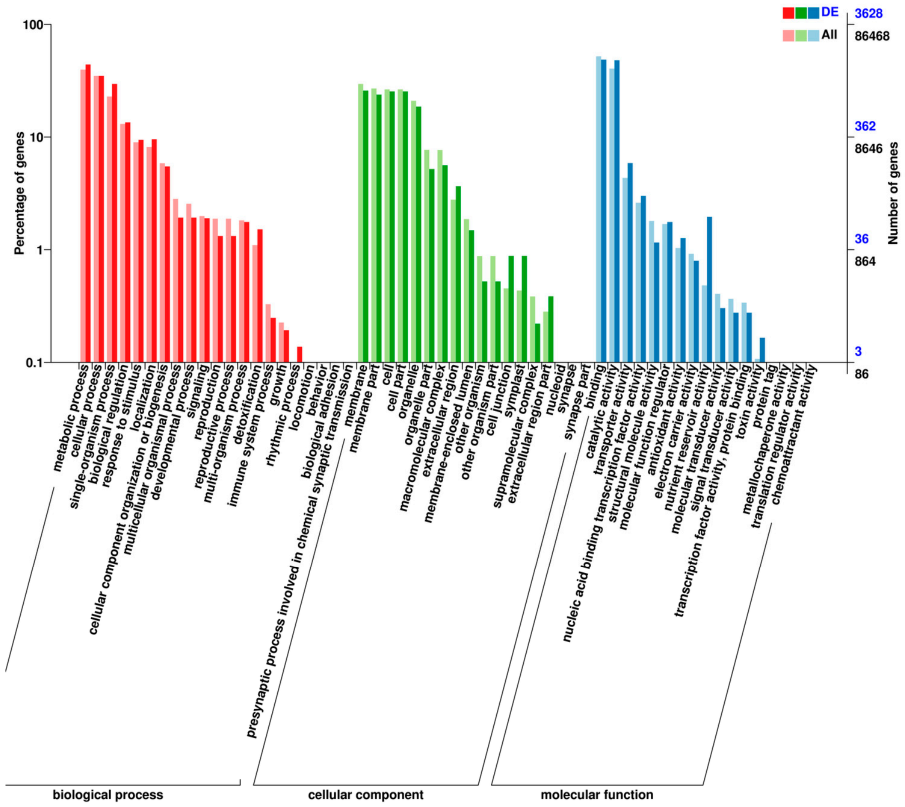


group4: ZMC vs II-ZMI;

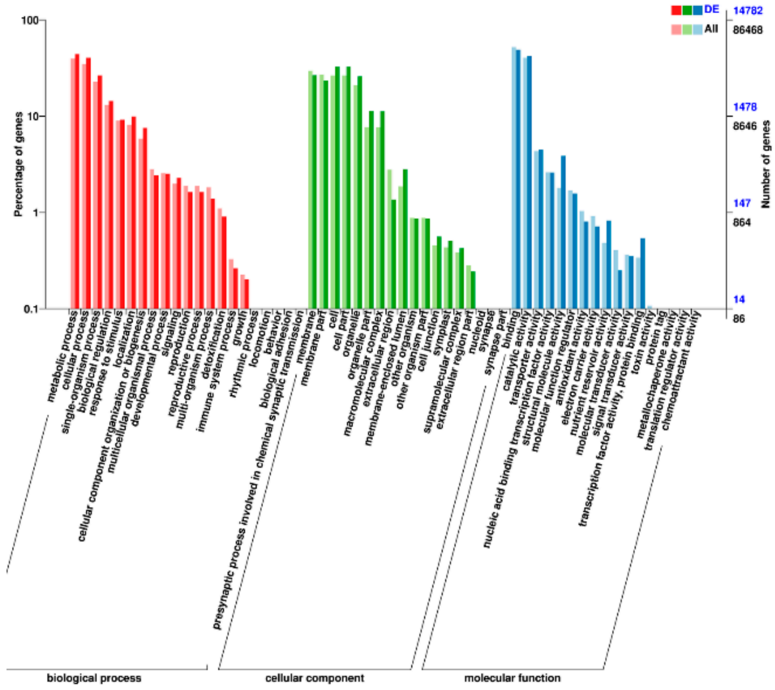


group5: III-YLC vs III-YLI;

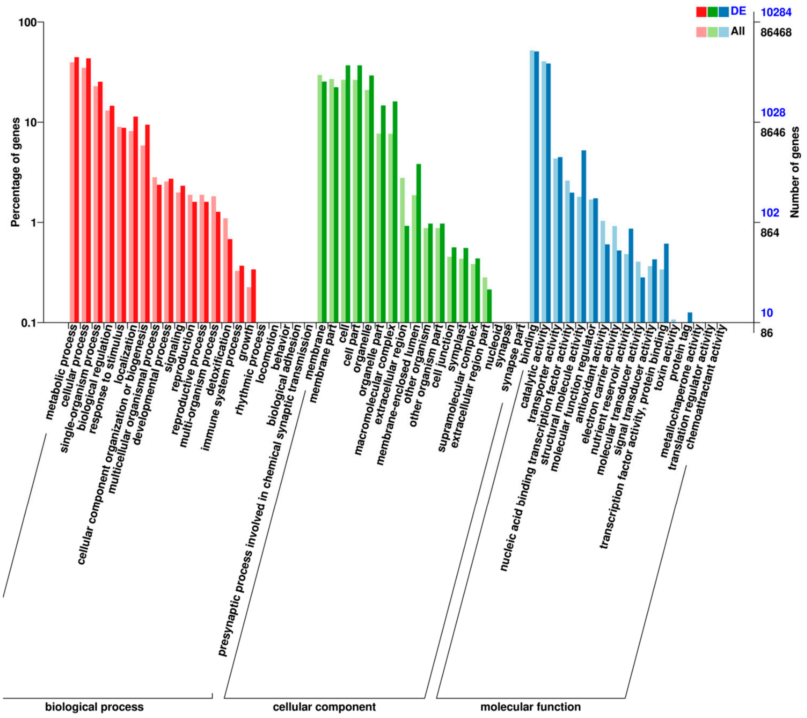


group6: III-ZMC vs III-ZMI

Figure S5 **GO annotation of Zhongmai 175 and Yili 053**

Note: The abscissa is the classification content of GO, the left side of the ordinate is the percentage of the number of genes, and the right side is the number of genes. group 1: I-YLC vs I-YLI; group2: I-ZMC vs I-ZMI;group3: II-YLC vs II-YLI; group4: ZMC vs II-ZMI; group5: III-YLC vs III-YLI; group6: III-ZMC vs III-ZMI

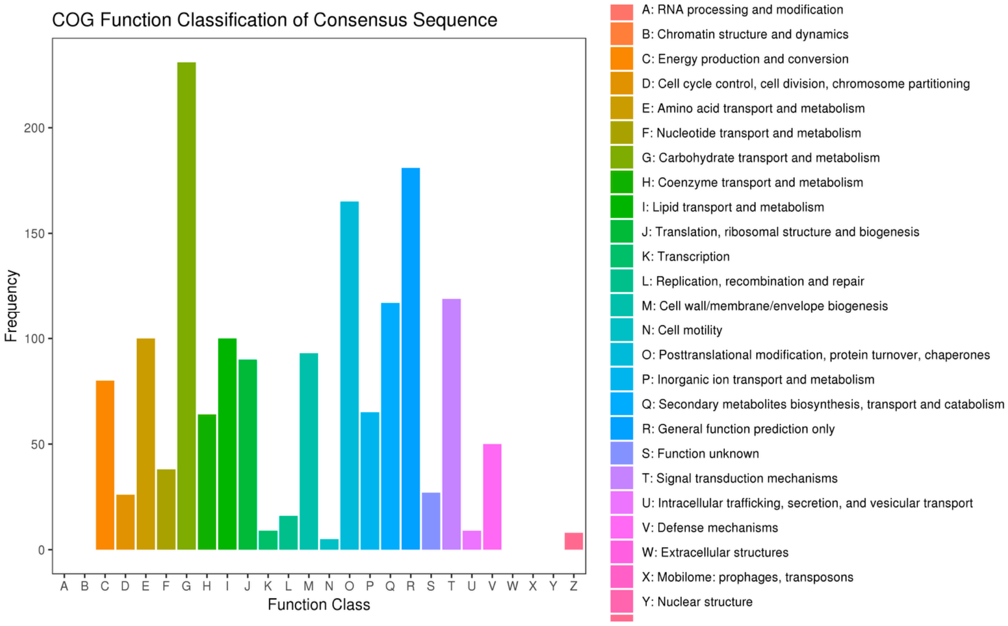


group 1: I-YLC vs I-YLI;

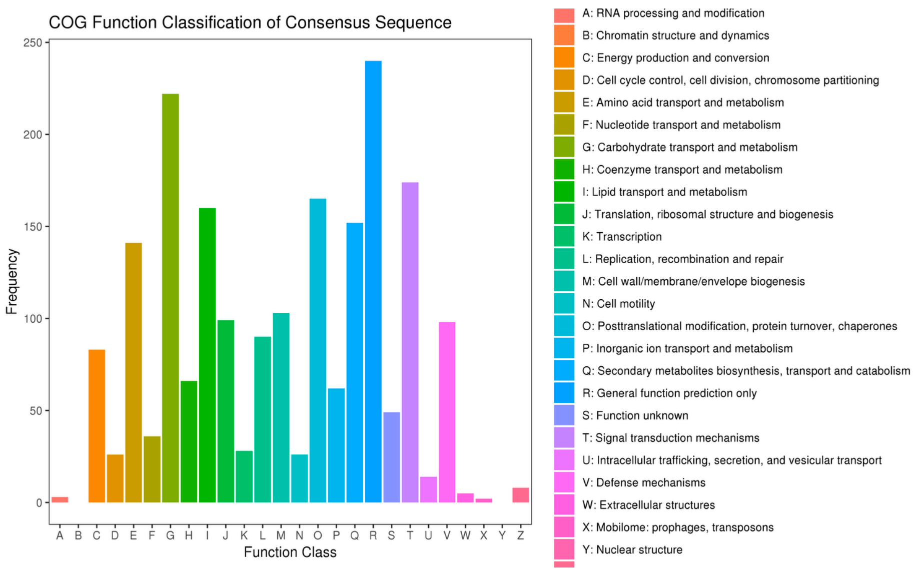


group2: I-ZMC vs I-ZMI;

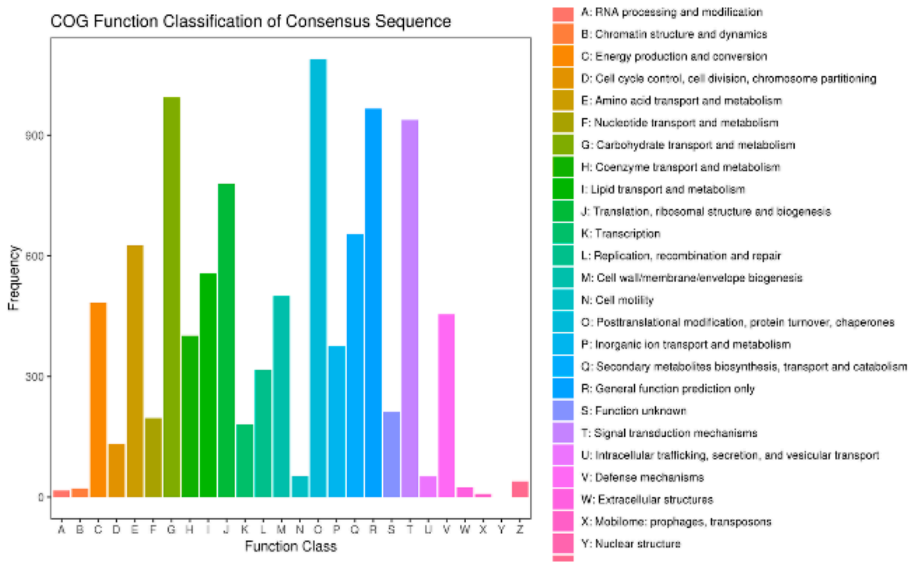


group3: II-YLC vs II-YLI;

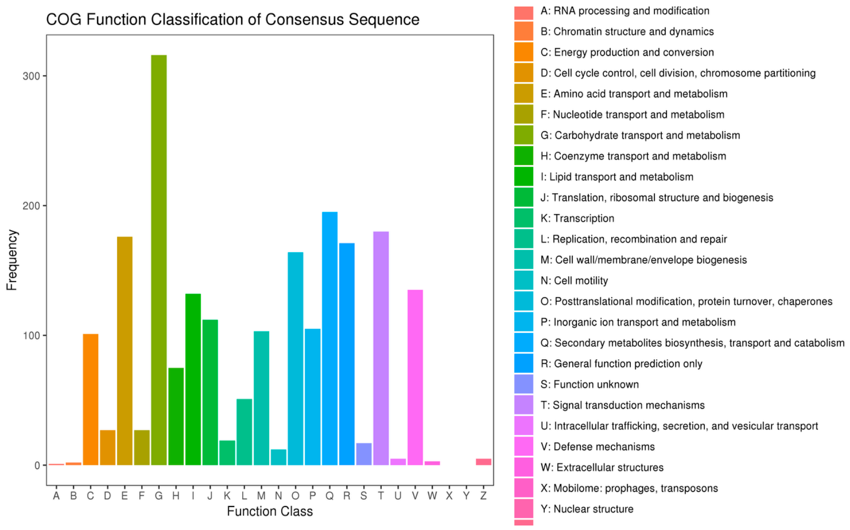

group4: II-ZMC vs II-ZMI;

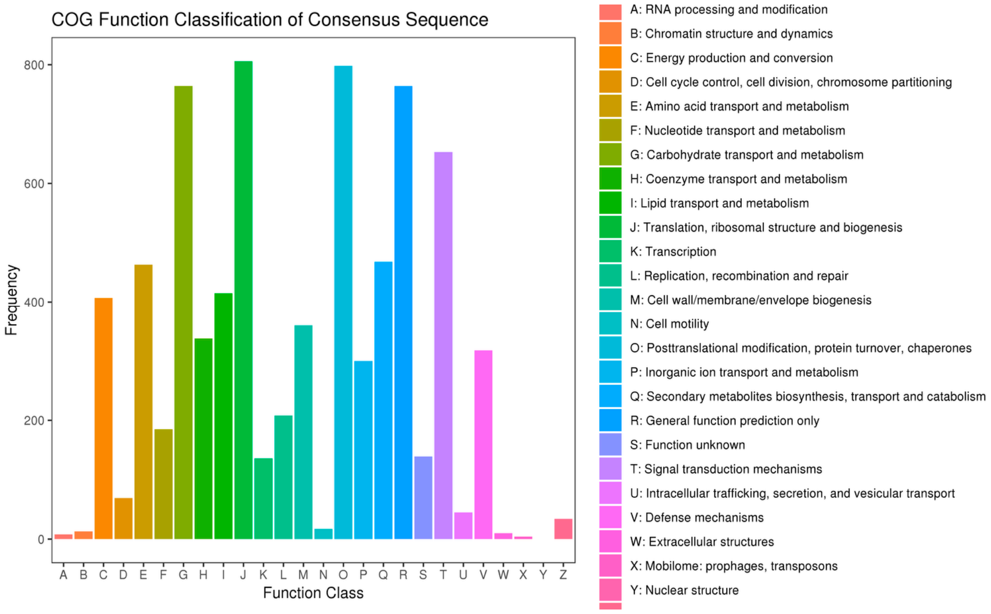


group5: III-YLC vs III-YLI;

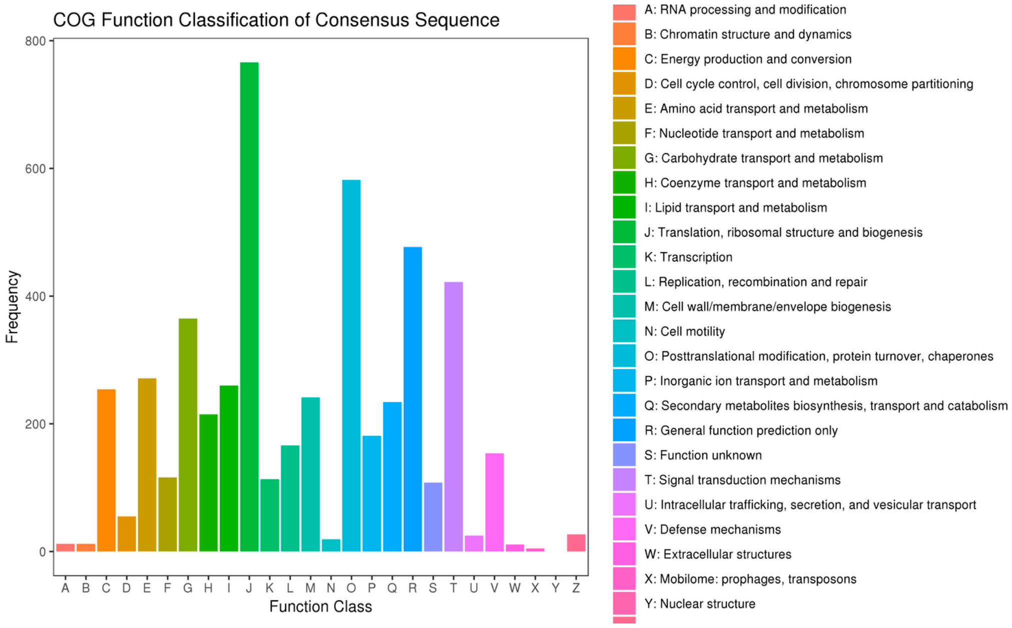


group6: III-ZMC vs III-ZMI

Figure S6 COG annotation of Zhongmai 175 and Yili 053

Note: The abscissa is the classification content of COG, and the ordinate is the number of genes. group 1: I-YLC vs I-YLI; group2: I-ZMC vs I-ZMI;group3: II-YLC vs II-YLI; group4: ZMC vs II-ZMI; group5: III-YLC vs III-YLI; group6: III-ZMC vs III-ZMI
